# Integrating Tenascin-C protein expression and 1q25 copy number status in pediatric intracranial ependymoma prognostication: A new model for risk stratification

**DOI:** 10.1371/journal.pone.0178351

**Published:** 2017-06-15

**Authors:** Felipe Andreiuolo, Gwénaël Le Teuff, Mohamed Amine Bayar, John-Paul Kilday, Torsten Pietsch, André O. von Bueren, Hendrik Witt, Andrey Korshunov, Piergiorgio Modena, Stefan M. Pfister, Mélanie Pagès, David Castel, Felice Giangaspero, Leila Chimelli, Pascale Varlet, Stefan Rutkowski, Didier Frappaz, Maura Massimino, Richard Grundy, Jacques Grill, on behalf of the SIOP Ependymoma Biology Working Group BIOMECA (BIOlogical Markers for Ependymomas in Children and Adolescents)

**Affiliations:** 1 Université Paris-Sud, Gustave Roussy, CNRS UMR 8203 "Vectorologie et Thérapeutiques Anticancéreuses", Villejuif, France; 2 Département de Neuropathologie, Hôpital Sainte-Anne, Paris, France; 3 Departamento de Patologia, Universidade Federal do Rio de Janeiro, Rio de Janeiro, Brazil; 4 Departement de Biostatistique et Epidemiologie, Gustave Roussy, Cancer Campus, Grand Paris, Villejuif, France; 5 CESP Centre for Research in Epidemiology and Population Health, INSERM U1018, Paris-Sud Univ., Villejuif, France; 6 Children’s Brain Tumour Research Network (CBTRN), Royal Manchester Children’s Hospital, Manchester, United Kingdom; 7 The Centre for Paediatric, Teenage and Young Adult Cancer, Institute of Cancer Sciences, The University of Manchester, Manchester, United Kingdom; 8 Institute of Neuropathology, University of Bonn Medical Center, Bonn, Germany; 9 Department of Paediatric Hematology and Oncology, University Medical Center Hamburg-Eppendorf, Hamburg, Germany; 10 Division of Pediatric Hematology and Oncology, Department of Pediatrics and Adolescent Medicine, University Medical Center Goettingen, Goettingen, Germany; 11 Division of Paediatric Neurooncology, German Cancer Research Center (DKFZ) and Department of Paediatric Oncology, Heidelberg University Hospital, Heidelberg, Germany; 12 Clinical Cooperation Unit Neuropathology, German Cancer Research Center (DKFZ), Heidelberg, Germany; 13 Genetics Unit, Pathology Department, Ospedale S. Anna, Como, Italy; 14 Université Sorbonne Paris Cité, Paris, France; 15 Département de Cancérologie de l'Enfant et de l'Adolescent, Gustave Roussy, Villejuif, France; 16 Department of Radiology, Oncology and Anatomo-Pathology, Sapienza University, Roma, Italy; 17 IRCCS Neuromed, Pozzilli, Isernia, Italy; 18 Institut d'Hématologie-Oncologie Pédiatrique, Lyon, France; 19 Paediatric Unit, Fondazione Istituto Di Ricovero e Cura a Carattere Scientifico, Istituto Nazionale dei Tumori, Milano, Italy; 20 The Children's Brain Tumour Research Centre, University of Nottingham, Nottingham, United Kingdom; Universidad de Navarra, SPAIN

## Abstract

**Purpose:**

Despite multimodal therapy, prognosis of pediatric intracranial ependymomas remains poor with a 5-year survival rate below 70% and frequent late deaths.

**Experimental design:**

This multicentric European study evaluated putative prognostic biomarkers. Tenascin-C (TNC) immunohistochemical expression and copy number status of 1q25 were retained for a pooled analysis of 5 independent cohorts. The prognostic value of TNC and 1q25 on the overall survival (OS) was assessed using a Cox model adjusted to age at diagnosis, tumor location, WHO grade, extent of resection, radiotherapy and stratified by cohort. Stratification on a predictor that did not satisfy the proportional hazards assumption was considered. Model performance was evaluated and an internal-external cross validation was performed.

**Results:**

Among complete cases with 5-year median follow-up (n = 470; 131 deaths), TNC and 1q25 gain were significantly associated with age at diagnosis and posterior fossa tumor location. 1q25 status added independent prognostic value for death beyond the classical variables with a hazard ratio (HR) = 2.19 95%CI = [1.29; 3.76] (p = 0.004), while TNC prognostic relation was tumor location-dependent with HR = 2.19 95%CI = [1.29; 3.76] (p = 0.004) in posterior fossa and HR = 0.64 [0.28; 1.48] (p = 0.295) in supratentorial (interaction p value = 0.015). The derived prognostic score identified 3 different robust risk groups. The omission of upfront RT was not associated with OS for good and intermediate prognostic groups while the absence of upfront RT was negatively associated with OS in the poor risk group.

**Conclusion:**

Integrated TNC expression and 1q25 status are useful to better stratify patients and to eventually adapt treatment regimens in pediatric intracranial ependymoma.

## Introduction

Ependymoma is the second most common malignant brain tumor in children. Half of the cases are diagnosed before the age of 5, two thirds arising in the posterior fossa. This disease comprises several entities, each with its own molecular pathogenesis, strongly influenced by age and location [1–7]. While supratentorial ependymomas are driven by specific translocations [5,6], infratentorial ependymomas are not and can be distinguished by their DNA methylation pattern [4,7]. Albeit molecularly heterogeneous, ependymomas share common biological and phenotypic characteristics, beyond histological features, for example Notch-1 pathway activation [2] or putative cell of origin [3]. The latest WHO classification update has individualized one of these entities, i.e the supratentorial ependymomas with RELA fusion, considering that the other subgroups could not be distinguished based on standard histology and molecular pathology [8]. Despite their grouping into 9 different entities in the latest publication [7], all ependymomas are actually still treated with the same protocol irrespective of their location. Pediatric ependymomas currently represent a therapeutic challenge, being incurable in at least one third of the cases despite multimodal therapy. However, some children can be cured without recourse to radiotherapy [9,10], while other will experience recurrence regardless of the use of optimal radiotherapy [11]. The extent of resection has been regularly found as the most important prognostic factor [9–11]. Several prognostic biomarkers for ependymoma have been identified in single reports but none of them has been validated prospectively for treatment stratification [12]. Grading according to the current World Health organization (WHO) classification has proved difficult to standardize [13] but has shown prognostic impact in some studies [11,13,14].

Previous studies in pediatric ependymoma reported, at recurrence, frequent gains of chromosome 9q33-34 region, i.e. the genomic region of *NOTCH1* and *Tenascin-C* (TNC), associated with the overexpression of TNC [2,15,16]. TNC is a large hexameric extracellular glycoprotein, with little or no expression detected in healthy adult tissues, and a known Notch-1 target. It is transiently re-expressed upon brain injury and down regulated after tissue repair is complete. TNC is involved in the generation of neural stem-cell niches, modulates matrix-cell interactions and in several types of cancer has been associated with increased vascularity, decreased survival and short time to relapse [17]. Evidence also supports its key role in the maintenance of a metastatic “niche” that would allow for the survival of disseminated tumor cells by activating NOTCH and WNT pathways [18]. TNC expression by immunohistochemistry (IHC) has been shown, specifically in ependymomas, to be associated with higher grade [15] and inferior event-free survival in small retrospective series [16,19]. Among two prognostic molecular groups of posterior fossa ependymoma identified, tumors from the group with poor prognosis were more frequently positive for TNC [4]. TNC expression is also more frequent in ependymomas of children than in those of adults [4,15].

Many studies have also reported chromosome 1q gain to be associated with worse prognosis in ependymoma but neither a candidate gene at 1q nor a definite biological explanation has been clearly identified so far [14,20–22].

Extent of resection and radiotherapy are the most important prognostic factors whatever the location or the subtype of the ependymoma [9–12]. The aim of this study was to provide a prognostication tool for all intracranial ependymomas that could be used to stratify every patient enrolled in an international trial. Biological prognostic markers, TNC and 1q25 gain, were added to the clinical and therapeutic parameters to improve the predictive accuracy of this prognostication tool.

## Materials and methods

### Patients

From the SIOP Ependymoma Biology Working Group BIOMECA (BIOlogical Markers for Ependymomas in Children and Adolescents), 595 patients from 5 national trial cohorts (France (FR) (n = 93), United Kingdom (UK) (n = 105), Italy (IT) (n = 62), Germany GPOH HIT 2000 trial (n = 139), and Heidelberg group (n = 196)) were identified. All patients included in the study were under 18 years, had a histologically confirmed newly diagnosed ependymoma that was centrally reviewed nationally according to WHO 2007 guidelines before selection of the patient samples for confection of tissue microarray (TMA) blocks. Patients without clinical records of treatment and comorbidities and without sufficient follow-up were excluded from the final analysis. All patients were treated by surgery. Upfront adjuvant radiotherapy (RT) +/- chemotherapy (CT) was administered for patients aged older than 3 and 5 years according to the country and regardless the extent of resection. Patients under 3–5 years were treated by chemotherapy as first line treatment. Treatments were defined by the national protocols listed in Section A in S1 File.

The studies were approved by the internal review boards of the sponsoring institutions in each country according to the regulation in place at the time of the conduct of the clinical study (see the initial publications of the trials in which the patients were enrolled, Section A in S1 File). Informed consent for these studies was obtained from the parents and guardians within the frame of a clinical research protocol when applicable or within a dedicated study for scientific purpose. (See S2 File for an example of the consent signed by the family of French patients).

### Specimen characteristics

Analyses were performed in formalin fixed paraffin embedded ependymoma samples from patients at first surgery before CT or RT, included in TMA blocks (Section A in S1 File).

### Assay methods

Preliminary studies in the consortium and extensive literature review led us to choose TNC and 1q25 to be evaluated as prognostic biomarkers in this collaborative endeavor [12]. TNC IHC was performed according to techniques described in Section A in S1 File. As previously described by Puget and coworkers [2], TNC IHC in ependymoma stained the extracellular matrix, and was generally not observed in individual cells, neither in the nucleus nor in the cytoplasm. Two main patterns (perivascular and intercellular) or a combination of both were observed (Fig A in S3 File). In some cases, TNC staining was heterogeneous within different regions of a same tumor. Immunohistochemical staining for TNC was scored based on staining intensity, as follows: 0: no staining; 1: weak staining; 2: moderate to strong staining (Fig A in S3 File). Scoring was based on most positive areas. For statistical analyses, moderate and strong staining was considered as overexpression (positive), compared to absent and weak staining (negative). Immunostains for TNC were performed using the same techniques and scored independently using the proposed scheme described above, by three observers. Reproducibility of staining and scoring for TNC was tested in the UK cohort by two independent observers, blindly, with excellent reproducibility (kappa = 0.91) (Section A in S1 File).

Chromosome 1q25 status was also studied on the same TMA material using FISH techniques (France, UK, Heidelberg), or on whole slides (IT) as previously described [19,20]. Cases from GPOH, had their 1q25 status analyzed by multiplex ligation-dependent probe amplification (MLPA) employing the SALSA MLPA P303 probemix (MRC Holland, Amsterdam, the Netherlands) (Section A in S1 File).

RELA-fusion positive supratentorial ependymomas were identified by one of the recognized methods to detect these fusions, i.e. FISH [5], RNAseq [6] or immunohistochemistry [5], depending on the material available and the cohort (Section A in S1 File).

### Study design

We collected all data concerning patients from the 4 countries included in various trials (Section A in S1 File) [9,10,22,23,24] and from one single center previously used for biomarker discovery [4]. TMA slides included tumor tissue appropriate to analyze TNC and 1q25 gain for most patients (Fig B in S3 File) and were used for IHC and FISH, respectively.

The median follow-up was estimated using the reverse Kaplan-Meier method. The endpoint was overall survival (OS), defined as the time from the date of diagnosis to the date of death from any cause. Survivors were censored at the date of their last follow-up. The cut-off date of this analysis was January 1st, 2009.

### Statistical analysis

The baseline characteristics (sex, age at diagnosis (<, ≥ 36 months), tumor location (posterior fossa, supratentorial), grade (II, III), extent of resection (incomplete, complete), upfront adjuvant RT, RELA-fusion (negative, positive) and the 2 markers (TNC and 1q25 gain) were described overall and by cohort. The association between the 2 markers (TNC and 1q25 gain) and the covariates was tested after adjusting for cohort (Cochran-Mantel-Haenszel test). The association with OS was tested using the log rank test comparing the unadjusted survival Kaplan-Meier curves. We reported 5-year OS and its 95% confidence interval (CI) estimated using Rothman’s method. The core model was a multivariable Cox model stratified by cohort and including age, tumor location, grade, extent of resection and treatment. This selection was based on established clinical knowledge. Sex was not a candidate variable. The prognostic value of each marker (TNC and 1q25 gain) was evaluated in adding one at a time and both in the core model [25]. These models were compared using Akaike criterion (AIC) for goodness-of-fit and integrated AUC (iAUC) for discriminant ability. This latter is defined by the integral of Area Under Curve and we fixed a time interval of 3 years (value close to 1 indicate a good discrimination). The proportional hazards (PH) assumption was tested for the selected model using Schoenfeld residuals with a global test and the model was stratified by some covariates if needed. A list of clinical interactions pre-specified by the clinicians (including interaction with cohort to measure the between-cohort heterogeneity) was tested one at a time. Significant interactions were included in the model and the stability of the final model was evaluated using bootstrap resampling [26]. From the final model, we derived a prognostic score, its distribution was reported and risk groups with different prognosis were created using a non-data-driven method [27]. Calibration was evaluated by estimating the agreement between predicted and observed probability of death. The performance validation used the internal-external cross validation approach proposed by Royston et al. [28]. All analyses were conducted on complete cases. In addition, we also performed subgroups analyses (posterior fossa and supratentorial apart) to describe the patients’ characteristics and evaluated the association between the two markers (TNC and 1q25 gain) and OS, to justify the use of one single model to predict outcome on the entire population of pediatric intracranial ependymomas. The nominal alpha level, within the pooled analysis, was p = 0.05. We used SAS 9.3 (SAS Institute Inc., Cary NC) and R packages (survival, survAUC and rms) for statistical analyses. Results were reported according to the REMARK recommendations [25]. More details on statistical analyses performed are given in the appendix (Section B in S1 File).

## Results

### Patient description

From the 595 pediatric patients with intracranial ependymomas identified, 478 patients (FR (n = 64), UK (n = 88), IT (n = 28), GPOH (n = 134) and Heidelberg (n = 164)), with complete data (= 80%) including results for both TNC and chromosome 1q25 gain were selected for the principal analysis (Fig B in S3 File). Median follow-up was 5.0 years [range: 0.0; 17.0]. Patients were predominantly male (61%), older than 36 months (63%), with grade III histology (71%), with tumors located in posterior fossa (69%), and treated with radiotherapy as first line therapy (with or without chemotherapy) (65%) (Table A in S4 File). As expected, children older than 36 months received post-operative radiation therapy with or without chemotherapy (81%) more often than younger patients (38%) (p<0.0001). Patients not irradiated at diagnosis were systematically irradiated at the time of relapse. The five-year OS of the entire population was 71%, not significantly different in the 5 cohorts (logrank test p-value = 0.26) (Fig C in S3 File). The median overall survival was 9.94 years with a minimum value for the FR cohort (7.66 years).

The baseline characteristics were comparable with those of patients either without material for TNC and/or chromosome 1q25 gain analysis (n = 91) or with material but missing clinical characteristics (n = 23) (Table B in S4 File). The following analyses were based on the complete data set (n = 470) excluding 6 patients with missing extent of resection and 2 with missing information on treatment.

### Association between Tenascin-C, 1q25 gain and covariates

Positivity for TNC was significantly more common in patients under 36 months (76% vs 45%, p<0.0001) and in posterior fossa tumors (69% vs 30%) (p<0.0001), while 1q25 gain was significantly more common in older patients (22% vs 13%, p<0.01) and in posterior fossa tumors (21% vs 13%, p<0.05). The 2 markers were not correlated (p = 0.79) (Table C in S4 File). None of these two biomarkers was correlated with RELA status (Table C in S4 File).

### Univariate analysis

**Fig 1:**
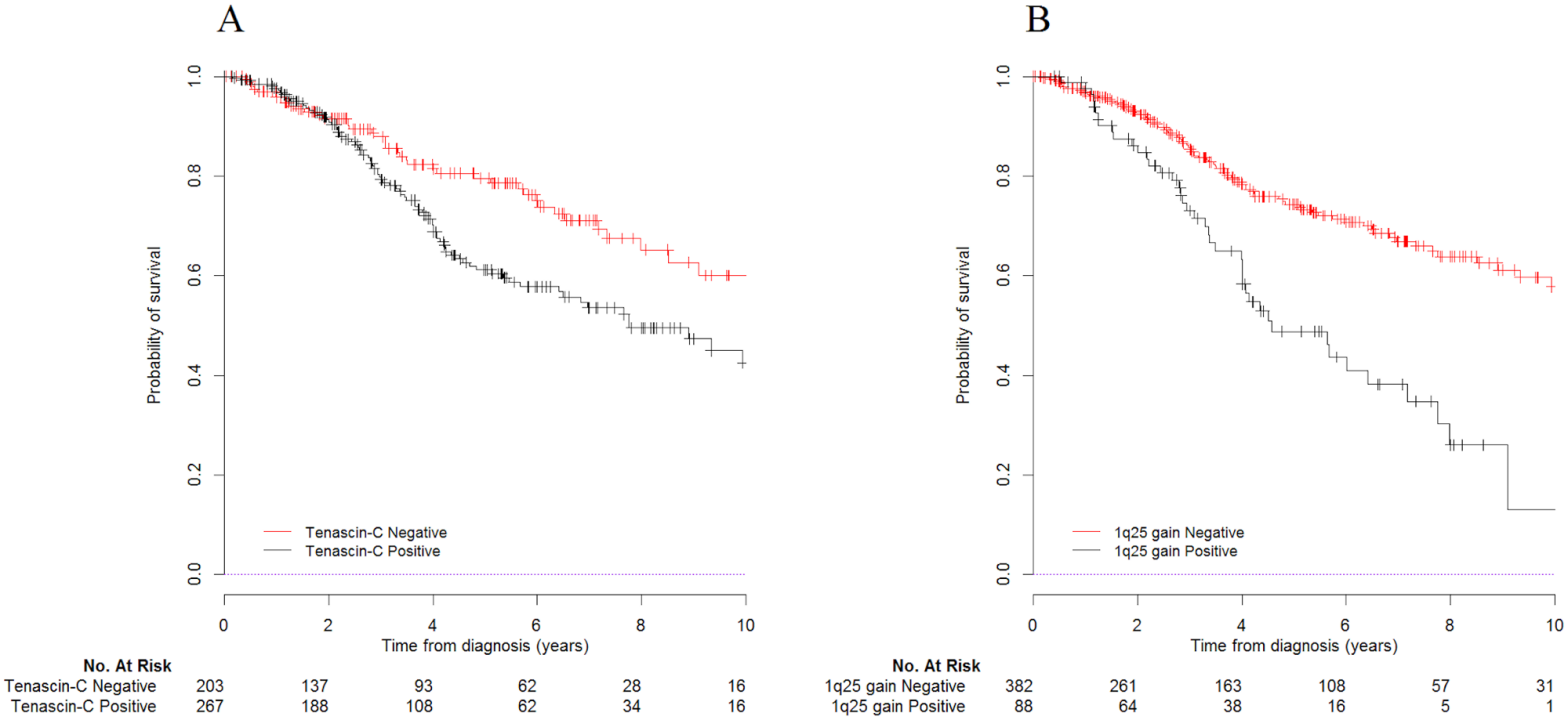
Kaplan-Meier-based overall survival curves according to Tenascin-C (negative (43%), positive (57%)) (A) and 1q25 gain (negative (81%), positive (19%)) (B) (n = 470). The hazard ratios (HR) and 95% confidence intervals, estimated through a univariate Cox model stratified by cohort, were for TNC: HR_pos vs neg_ = 1.586 [1.105; 2.277] (p = 0.012) and for 1q25 gain: HR_pos vs neg_ = 2.490 [1.721; 3.605] (p<0.0001).

Twenty-eight percent (131/470) of patients died during follow up. Patients without TNC overexpression had a longer OS (median: 12.5 years 95%CI = [9.1; NE]) compared to patients with TNC overexpression (median: 7.8 y [6.4; NE]) (p = 0.012) (Fig 1A). The 5-year OS was 79.6% [72.1; 85.5] and 61.2% [53.7; 68.2] in patients with tumors negative and positive for TNC, respectively. Similar results were observed for 1q25 gain with a median OS of 12.5 y [9.9; NE] and 4.6 y [4.0; 7.8] in patients with negative and positive status, respectively (p<0.0001) (Fig 1B). The 5-year OS was 74.3% [68.5; 79.4] and 48.8% [36.7; 61.0] in patients with negative and positive 1q25 gain status, respectively.

### Model building

From the core model using clinical variables and grading (model 1), we constructed 3 models by adding TNC alone (model 2), 1q25 gain alone (model 3) and the 2 markers (model 4). Model 3 showed a better goodness-to-fit, i.e lower AIC (AIC = 969,7) and a better discriminant ability, ie higher iAUC (iAUC = 0.70) than model 1 and 2 AIC = 992.8 and 991.0, iAUC = 0.63 and 0.64, respectively) (Table D in S4 File). Model 4 with TNC and 1q25 did not give additional information with a difference between AIC lower than 3 (AIC: 967.8, iAUC = 0.70) even if TNC was marginally significant with HR = 1.49 [0.99; 2.22] (p = 0.051). In model 3, the hazard ratio (HR) for patients with positive 1q25 gain was HR_pos vs neg_ = 2.83 [1.93; 4.16] (p<0.0001). Grade and extent of resection were also significantly associated with OS (p<0.05). The global test of PH assumption was significant (p = 0.0055) with a high violation of PH assumption by RT (p = 0.0139). The association of upfront RT with overall survival is time-dependent; this means that the advantage of receiving upfront RT is only significant during the first 3 years after diagnosis (data not shown). After stratification on RT covariate as a time-dependent variable, the global test of PH assumption was no longer significant (p = 0.338). This stratification enables to define a baseline hazard related to upfront RT and also having a more stable model regarding the correlation between upfront RT and age. The results are reported in the second column of Table E in S4 File.

The next step in building the model was to evaluate some pre-specified interactions listed in Table C in S4 File. No heterogeneity of the effect of TNC and 1q25 gain across trials was observed. The significant interactions (age x grade, tumor location x TNC and tumor location x 1q25) were included and only tumor location x TNC (p = 0.014) was retained in the final model (Table E in S4 File). This model leads to a better AIC compared to the model without interaction (817.4 vs 823.8) with a slightly better discriminant ability (iAUC = 0.70 vs 0.68). In terms of HR, a statistically significant deleterious effect of positive TNC was observed in patients with posterior fossa tumors (HR_pos vs neg_ = 2.19 [1.29; 3.76] (p = 0.004) while no significant effect was observed in patients with supratentorial tumors (HR_pos vs neg_ = 0.64 [0.28; 1.48] (p = 0.295) (interaction test p = 0.015). HR of 1q25 gain did not change substantially compared to the ones estimated from model 3 (HR_pos vs neg_ = 2.97 [1.99; 4.43] (p<0.0001). RELA-fusion was not included in the final model because of the exclusion of 45% of data (RELA is only defined in the supratentorial ependymomas).

### Pediatric Intracranial Ependymomas Score (PIES), risk stratification and calibration

**Fig 2:**
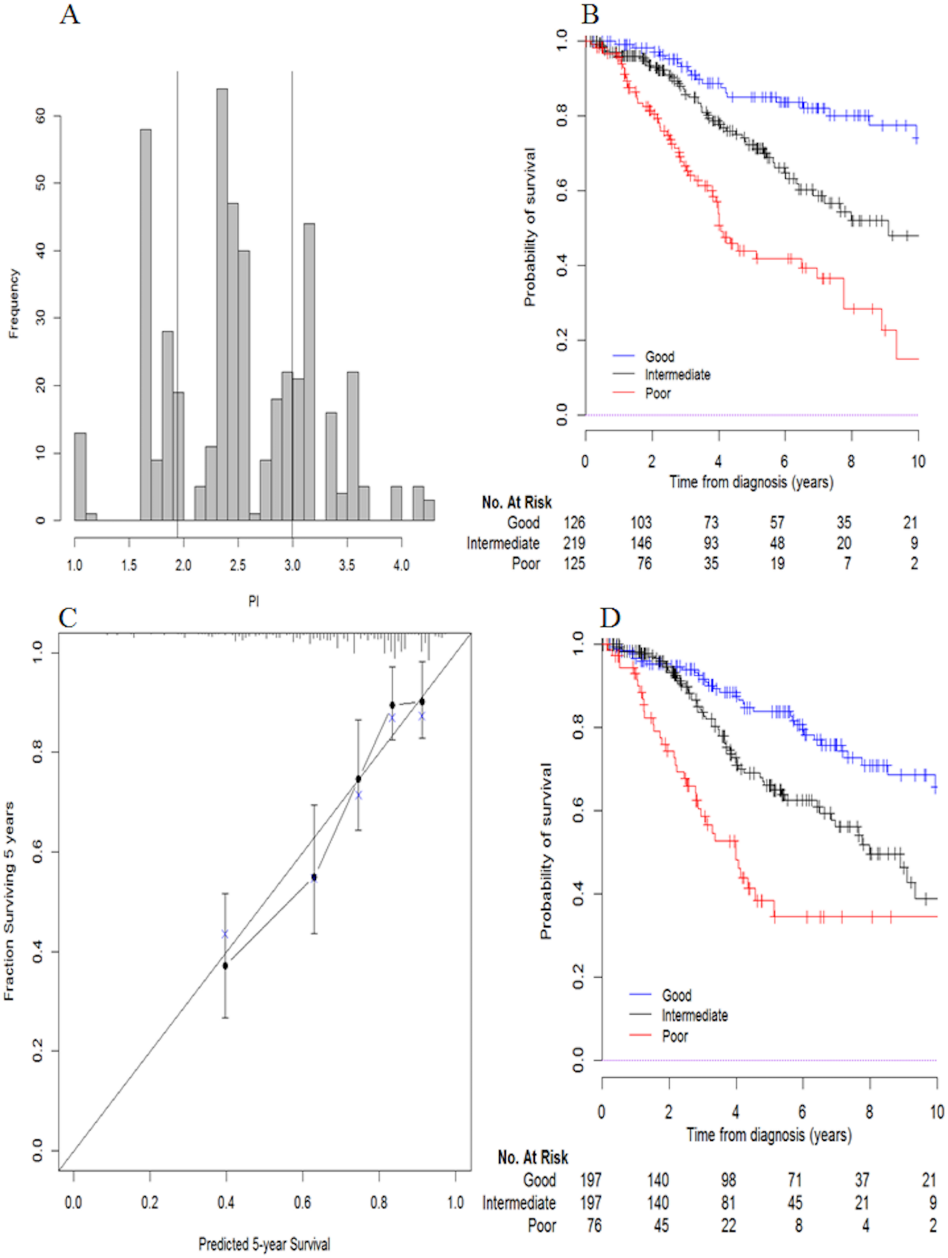
A) Histogram of Pediatric Intracranial Ependymomas Score (PIES), B) Kaplan-Meier-based overall survival curves of 3 risk groups, C) Agreement between predicted and observed probability of death at 5 years and D) Kaplan-Meier-based overall survival curves of 3 risk groups using internal-external cross-validation approach.

From the final model (Table E in S4 File), we developed a prognostic score called Pediatric Intracranial Ependymomas Score (PIES) for OS with a mean (standard deviation) of 2.52 (0.67) (Fig 2A). PIES was calculated, for each patient, as a weighted sum of the covariates in the final model, where the weights are the regression coefficients (Table 1). Three risk groups were defined by cut-points placed at the 27 and 73 percentile of the PIES (cut-points = 1.943 and 2.991): poor risk group includes patients with grade III (93%), incomplete extent of resection (80%), positive TNC (82%) and 1q gain (48%), good risk group includes patients ≥36 old months (78%), with grade II (68%), complete extent of resection (77%) and absence of 1q25 gain (100%).

**Table 1:** Regression coefficients of Pediatric Intracranial Ependymomas Score (PIES).

Prognostic factor		B
Age at diagnosis	≥ 36 months vs <36months	-0.08818
Tumor location	Posterior fossa vs supratentorial	0.61200
Grade	III vs II	0.66265
Extent of resection	Complete vs Incomplete	-0.57949
Tenascin C	Posterior fossa: Positive vs Negative	0.78724
	Supratentorial: Positive vs Negative	-0.44741
1q25 gain	Positive vs Negative	1.08820

PIES was calculated, for each patient, as follows: *PIES* = *β*_1_ *I*(*age* ≥ 36) + *β*_2_ *I*(*tumor location* = *supratentorial*) + *β*_3_ *I*(*grade* = *III*) + *β*_4_ *I*(*extent of resection* = *complete*) + *β*_5_ *I*(*Tenascin C* = *positive,tumor location* = *posterior fossa*) + *β*_6_ *I*(*Tenascin C* = *positive,tumor location* = *supratentorial*) + *β*_7_ *I*(1*q gain* = *positive*)

*with I*(*x*) = 1 *if x is true*, 0 *otherwise*

and a patient is classified in one risk group as follows:

*if PIES* < 1.943 (27*th percentile*) *then risk* = *good*

*else if* 1.943 ≤ *PIES* ≤ 2.991 (73*th percentile*) *then risk* = *intermediate*

*else if PIES* > 2.991 *then risk* = *poor*

Fig 2B shows the Kaplan-Meier estimation of OS for the 3 risk groups with a good separation: HR_intermediate vs good_ = 2.39 [1.44; 3.97] and HR_poor vs good_ = 5.36 [3.21; 8.96]. The 5-year OS was 85.1% [76.5; 90.9] in the good prognosis group (n = 126), 72.3% [64.1; 79.3] in the intermediate group (n = 219) and 44.0% [33.2; 55.4] in the poor prognosis group (n = 125). No heterogeneity of the risk group (poor, intermediate, good) was observed across national cohorts (p = 0.146) and the separation is globally well maintained across the cohorts. The agreement between predicted and observed probability of death at 5 years (calibration) is represented in Fig 2C with groups of approximately 80 patients to have reliable estimate. The figure shows an acceptable calibration. We observed a significant association between upfront RT and OS in poor risk group (HR = 0.377 [0.158, 0.898] (p = 0.028) while no significant difference is observed in good risk group (HR = 2.074 [0.611, 7.035]; p = 0.242) and intermediate risk group (HR = 1.042 [0.486, 2.233]; p = 0.916) (Fig D in S3 File). HRs of upfront RT were estimated from a Cox model stratified on cohort and controlling for age, tumor location, grade, extent of surgery, TNC, TNC x tumor location interaction and 1q25 gain.

### Model validation

An internal-external cross validation approach was used to validate our PIES [27]. After omitting one cohort, fitting the model (Table E in S4 File) on 4 other cohorts and calculating the 27 and 73 percentiles of PIES (to define the cut-offs), we calculated PIES for patients from the omitted cohort and classified them into good, intermediate or poor prognosis according to these cut-offs. After repeating these steps for each cohort, we can estimate the Kaplan-Meier OS curves for the 3 risk groups including all patients. Fig 2D shows a good discrimination between the three groups. We ended up model validation by calculating iAUC using the same approach. The values of iAUC (>0.62) estimated on independent cohort were good with small difference from the ones estimated on the training set. The discriminant ability appears to replicate well from the set of cohort omitting one (iAUC: from 0.67 to 0.73) and the remaining cohort (iAUC: 0.63 to 0.73).

### Posterior fossa and supratentorial subgroups

Although potential possible heterogeneity between these two biological entities has been captured by adding interaction terms between tumor localization and covariates for developing model in the pooled analysis, we described the patients’ characteristics and performed a multivariable analysis for these 2 entities, separately.

When the multivariable analysis was restricted to posterior fossa ependymomas, grade III, extent of resection, TNC immunopositivity and 1q25 gain were associated with OS (Table 2, See Table G in S4 File for description).

**Table 2:** Multivariable model for overall survival in patients with posterior fossa ependymomas (N = 325). The multivariable Cox regression model is stratified by cohort and radiotherapy^‡^.

Prognostic factors		Hazard Ratio	95% confidence interval	p-value
Age at diagnosis	<36months	1		0.1662
	≥ 36 months	0.685	[0.402; 1.170]	
Grade	II	1		0.0283
	III	1.710	[1.059; 2.761]	
Extent of resection	Incomplete	1		0.0043
	Complete	0.525	[0.338; 0.817]	
Tenascin-C	Negative	1		0.0184
	Positive	1.941	[1.118; 3.367]	
1q25 gain	Negative	1		0.0001
	Positive	2.491	[1.561; 3.976]	

^‡^: RELA is not evaluated in the posterior fossa

**Fig 3:**
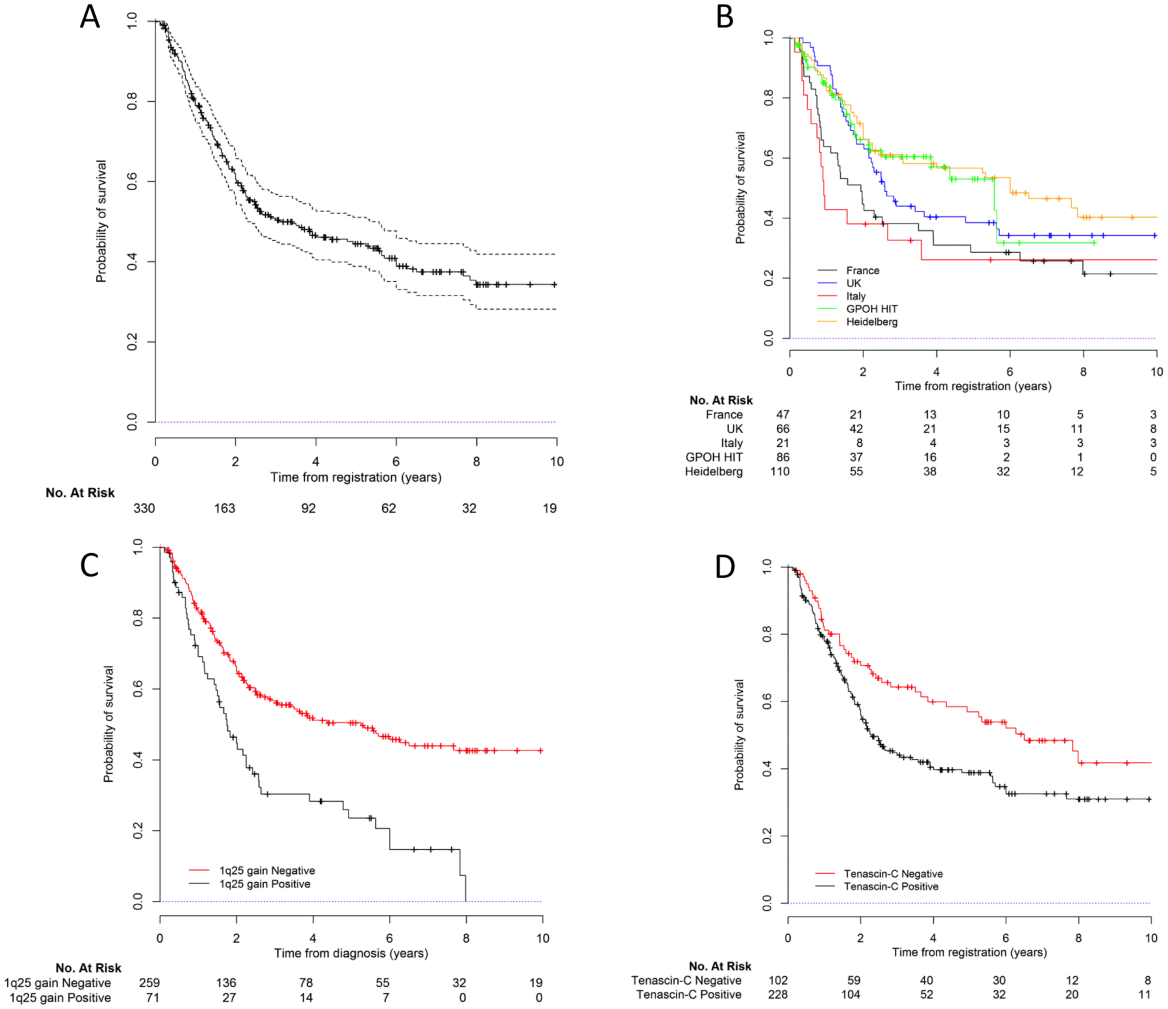
Survival curves for posterior fossa tumor patients. A) Global overall survival; B) Overall survival by cohort; C) by 1q status and D) by TNC expression.

Fig 3 shows the OS curves for the whole group of posterior fossa ependymoma, and according to cohort, 1q25 status and TNC immunopositivity.

When the multivariable analysis was restricted to supratentorial ependymomas, only 1q25 gain remained significantly associated with OS (Table 3, See Table H in S4 File for description).

**Table 3:** Multivariable model for overall survival in patients with supratentorial ependymomas (N = 145). The multivariable Cox regression model is stratified by cohort and radiotherapy.

Prognostic factors		Hazard Ratio	95% confidence interval	p-value
Age at diagnosis	<36months	1		0.1617
	≥ 36 months	2.881	[0.655; 12.680]	
Grade	II	1		0.0613
	III	4.787	[0.928; 24.676]	
Extent of resection	Incomplete	1		0.1871
	Complete	0.565	[0.242; 1.319]	
Tenascin-C	Negative	1		0.1149
	Positive	0.474	[0.188; 1.199]	
1q25 gain	Negative	1		0.0067
	Positive	3.261	[1.389; 7.658]	

When RELA-fusion status was added in the multivariable model, it was not retained as significant (Table 4):

**Table 4:** Multivariable model for overall survival in patients with supratentorial ependymomas with available RELA-fusion status (N = 72). The multivariable Cox regression model is stratified by cohort and radiotherapy.

Prognostic factors		Hazard Ratio	95% confidence interval	p-value
Age at diagnosis	<36months	1		0.1612
	≥ 36 months	4.281	[0.560; 32.752]	
Grade	II	1		0.1161
	III	8.835	[0.583; 133.789]	
Extent of resection	Incomplete	1		0.8723
	Complete	1.100	[0.344; 3.515]	
Tenascin-C	Negative	1		0.1811
	Positive	0.427	[0.122; 1.487]	
1q25 gain	Negative	1		0.0666
	Positive	3.586	[0.916; 14.032]	
RELA	Negative	1		0.5777
	Positive	0.669	[0.163; 2.750]	

**Fig 4:**
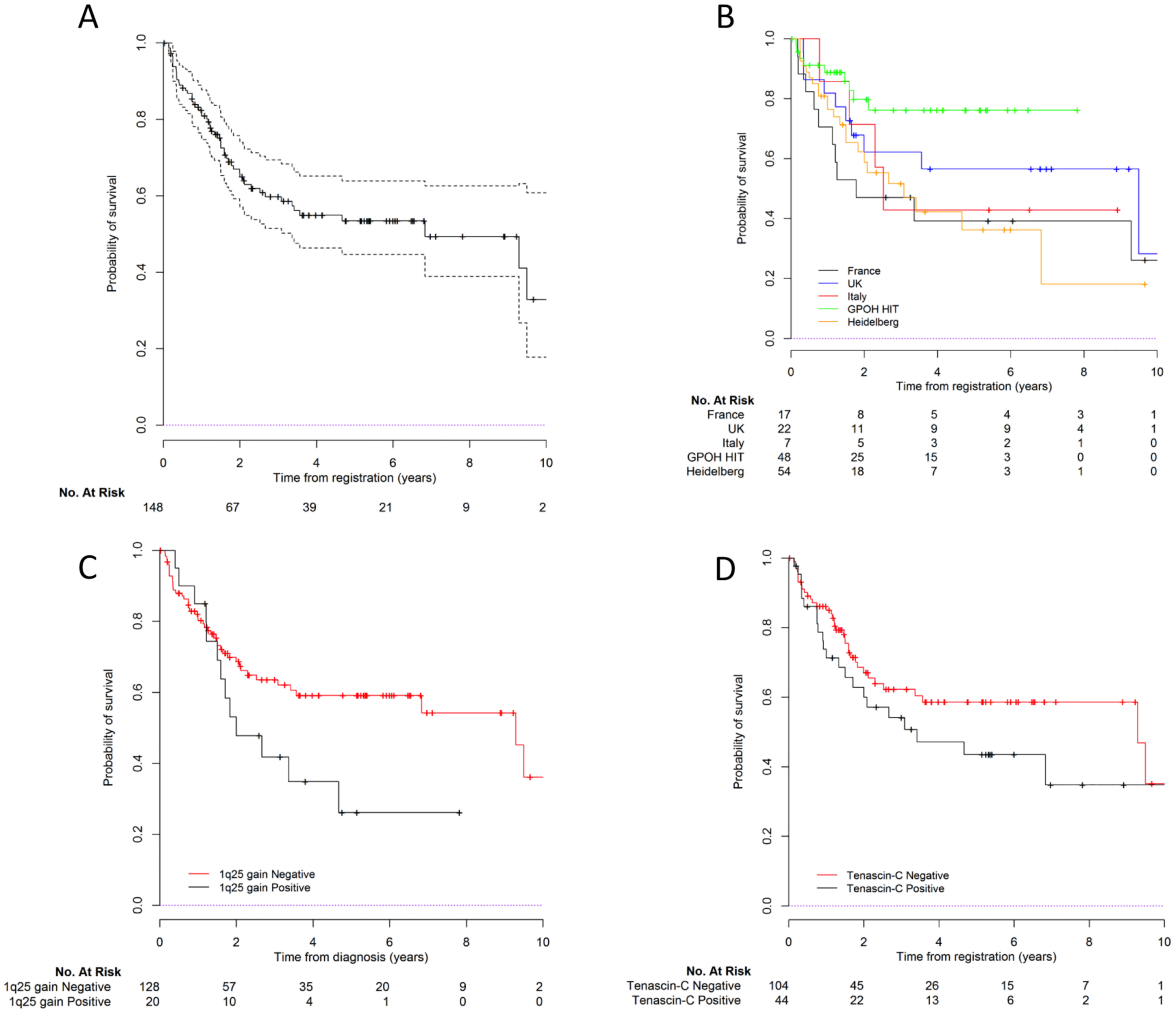
Survival curves for supratentorial tumor patients. A) Global overall survival; B) Overall survival by cohort; C) by 1q status and D) by TNC expression.

Fig 4 shows the OS curves for the whole group of supratentorial ependymoma, and according to cohort, 1q25 status and TNC immunopositivity.

## Discussion

This is the first study to propose an integrated score, combining clinical and pathological covariates with biomarkers for prognostication of pediatric ependymoma across multiple national cohorts. This unique and largest pooled analysis published so far allows to study interactions between covariates predicting overall survival. We choose to model the overall survival since progression-free survival would have been too much influenced by the initial treatment; indeed, young children were not treated with radiation and were therefore more prone to early relapses. In this respect, the association of upfront RT with OS could be specifically assessed since the various trials used different strategies with or without RT included in the first line treatment. Biomarkers chosen had been previously recognized but not completely validated. We showed that (i) the model performance including 1q25gain (model 3) is better than the models with no marker (model 1) and with TNC (model 2) and (ii) the model performance including both markers (model 4) did not improve substantially the performance of model 3. We, however, report that taking into account the interaction between TNC and tumor location (last column of Table E in S4 File) improved the performance of models 3 and 4. This is due to the fact that the prognostic effect of TNC was different according to tumor location. We decided to develop one model for all intracranial ependymoma and not 2 models (one for posterior fossa and one for supratentorial) in order to maximize the ability to study the interactions in the largest cohort possible. This approach was considered appropriate since treatment strategies are presently not stratified by location. When the analyses were restricted to the posterior fossa or supratentorial ependymomas, similar effect on overall survival were observed for 1q25 gain and TNC immunopositivity, but with limited power compared to the pooled population irrespective of the location.

Taking into account the major subtypes of ependymomas in each location, ie RELA-fusion positive or negative supratentorial tumors and PFA or PFB tumors, would also be of importance. Due to the retrospective nature of the study and the difficulty to obtain the methylation profile for all the samples, we could not incorporate it in the scoring. Moreover, this methylation profile may be difficult to obtain prospectively in every center because of costs and the recent change of the array version (450K to 850K) may need a re-validation of the results. Presently, there are two types of posterior fossa ependymomas defined by methylation profiling, PFA and PFB. These entities largely corresponding to pediatric and adult ependymomas, respectively, could also be distinguished by IHC as shown by Witt and coworkers [4]. Indeed, most PFA identified with methylation profiling (i.e 94%) were in fact positive for TNC while only 11% of PFB ependymomas were in fact positive for TNC. We could therefore assume that TNC IHC could be a simple surrogate for methylation profiling of PF ependymomas. The impact of TNC on overall survival is limited to the posterior fossa tumors in which its positivity is significantly more frequent.

The reproducibility of the IHC for TNC was validated in the study, including its scoring, and this has still to be proven for methylation studies. As the derived PIES score is a powerful tool to stratify the outcome of patients, it would be interesting to study in the future if the methylation profiling improves the performance of this prognostic score. Regarding supratentorial ependymomas, RELA-fusion status could be obtained in 72 out of 145 tumors. The presence of the RELA-fusion was correlated neither with TNC immunopositivity, nor with 1q25 gain. When the RELA-fusion status was incorporated in the multivariable model of overall survival in supratentorial ependymomas, it was not retained as significant besides 1q25 gain.

While controversial results have been reported on the prognostic significance of WHO histological grade in pediatric ependymoma [13,14,20], we found that histological grade III was significantly correlated with worse OS as reported by Merchant and coworkers [11] In our series, this prognostic effect remains homogeneous across cohorts (interaction p-value = p = 0.756). Despite a well-known heterogeneity of grading reported by different pathologists and cohorts,[13] in this large series grade remains a strong prognostic factor. Indeed, criteria used for grading are associated with tumor aggressiveness even if their reproducibility may vary among pathologists [13]. Thus, although the assignment of a given tumor to a given grade may be less reliable than other prognostic variables used in the model (e.g. location or age), the impact of the grade has still to be considered for prognostication in a multivariate approach.

A meta-analysis has shown that 1q gain is the most frequent genetic alteration in childhood ependymoma. Different studies report the gain of 1q as a marker of poor prognosis in ependymoma [14,20,22,23,29,30], and one publication has included part of the patients of the present series [21]. In the paper by Witt and colleagues including posterior fossa ependymoma from all ages, as observed for TNC, 1q gains had a higher occurrence in group A, and shared the association with worse prognosis. Interestingly, in the validation of the gene expression data performed on an independent cohort, patients from group A with 1q gains assessed by FISH exhibited no difference in survival compared with other group A patients, whose tumors did not display this aberration [4]. This is not surprising, if one considers that TNC is also overexpressed in group A patients and independently from 1q25 gain. In fact, only 19% of patients showed 1q25 gains while TNC overexpression was observed in 57%; consequently, the model incorporating the two risk factors was more effective to describe the prognosis of the whole population. In the recent study by Pajtler and coworkers [7], 1q gain was a strong prognostic factor across all subgroups of ependymomas, irrespective of their location.

Although a significant difference was observed between upfront RT and no upfront RT in high risk group only, caution about this finding is required due to possible bias because (i) this study was not designed to evaluate RT effect and (ii) even if the association between RT and overall survival was estimated from a multivariable model it is possible that confounders affecting both the administration of RT and overall survival were not captured even if we believe that their impacts are marginal. The finding that omitting to give radiotherapy as part of the first treatment was only detrimental for the high risk patients may challenge its systematic use in low-risk tumors, especially in young children.

Our data with simple and reproducible assays support the prospective assessment of these two biomarkers in clinical practice. They confirm, on a large multi-centric cohort of almost 500 children, the single center results from Austria where TNC and 1q25 gain were also shown to be prognostic in a series of 52 posterior fossa ependymomas [31]. IHC and FISH techniques are widely available as standard techniques in diagnostic neuropathology laboratories, and are already part of the regular assessment of other pediatric brain tumors such as medulloblastomas. The PIES score should be easily performed in current practice and represents a potential tool to stratify patients in randomized trials. In case new biomarkers would be identified, the same methodology would be applicable to see if their incorporation in the survival prediction model would improve its performance.

## Supporting information

S1 File Supplementary text. **—Section A.** Patients, Immunohistochemistry, 1q status assessment; **Section B.** Details of the statistical analyses.
(DOCX)

S2 File Informed consent sample (French patients). (DOCX)

S3 File Supplementary figures. **—Fig A. TNC immunostaining in pediatric ependymoma**. Upper panel: qualitative aspects of TNC staining: (A) Perivascular staining; (B) Perivascular and intercellular staining. Lower panel: TNC scoring: most positive areas were analyzed and scored for intensity of staining as shown. Only moderate and strong staining were considered as overexpression; **Fig B. Flow chart; Fig C. Kaplan-Meier-based overall survival curves overall** (dashed lines represent the 95% confidence bands) and by cohort (n = 478); **Fig D. Kaplan-Meier-based overall survival by radiotherapy, for good (A), intermediate (B) and poor (C) risk groups.**
(ZIP)

S4 File Supplementary tables. **—Table A.** Baseline characteristics, by cohort and for all patients; **Table B.** Patient and tumor characteristics for patients with and without TNC and 1q25 gain results; **Table C.** Correlation between Tenascin-C and 1q25 gain and baseline characteristics in all patients—complete cases analysis; **Table D.** Analysis of overall survival (OS) using a multivariable Cox regression model stratified by cohort in complete cases; **Table E.** Analysis of overall survival (OS) using a multivariable Cox regression model without and with interaction between TNC and tumor location stratified by cohort and radiotherapy in complete cases; **Table F.** P-values of pre-specified interaction terms; **Table G.** Baseline characteristics, by cohort and overall in posterior fossa patients; **Table H.** Baseline characteristics, by cohort and overall in supratentorial patients.
(ZIP)

S5 File Individual patient database from all cohorts, anonymized. (XLS)

S6 File Statement necker hospital brain tumor collection. (PDF)
