# Supplementary material for: Integrating Tenascin-C protein expression and 1q25 copy number status in pediatric intracranial ependymoma prognostication: A new model for risk stratification
Source: PLoS One. 2017 Jun 15;12(6):e0178351. doi: 10.1371/journal.pone.0178351 (PMC5472261; doi:10.1371/journal.pone.0178351)
Supplement: S1 File — —Section A. Patients, Immunohistochemistry, 1q status assessment; Section B. Details of the statistical analyses. (DOCX) [file pone.0178351.s001.docx]

**Supplementary data- Text**

**A: Patients, Immunohistochemistry, 1q status assessment**

Treatment protocols for the national clinical trial cohorts

| Country | Age limits | Protocol | Reference |
| --- | --- | --- | --- |
| France | <5 y | BBSFOP | Grill et al, J Clin Oncol 2001. |
| France | ≥5 y | RT only | Veelen-Vincent et al, J Neurosurg 2002. |
| Italy | <3 y | Infant AIEOP | Massimino et al, IJROBP 2011. |
| Italy | ≥3 y | HyperF-RT | Massimino et al, IJROBP 2004 |
| UK | < 3y | CNS9204 | Grundy et al, Lancet Oncol 2007 |
| UK | > 3y | CNS9904 | Ellison et al, J Neg Res Biomed 2011. |
| Germany | <3 y | HITSKK 87/92 | Timmermann et al, Radiat Oncol 2005. |
| Russia | <18 y | Burdenko | Witt et al, Cancer Cell 2011. |

Tenascin C: Immunohistochemistry

After depparafination 4 µm sections were submitted to antigen retrieval for 30 minutes at Citrate buffer ph 6.0 then incubated with primary antibody (TNC, mouse monoclonal antibody, clone E9, diluted at 1/50, Santa Cruz, ref SC 25328) at room temperature for 1 hour. The Vectastain Elite (Vector laboratories, ref. PK 6200) revelation kit used was used according to manufacturer’s instructions.

An automated technique was developed using the same antibody and dilution on Ventana Benchmark automated system, after antigen retrieval for 32 minutes at CC2 media, pH 6.0.

Reproducibility of the TNC IHC scoring

TNC staining techniques were performed in two different labs on and analysed blindly on TMA blocks from the UK cohort by two observers (FA, JPK) n=94 using the grading score proposed in S1 Figure. There was very good reproducibility with a kappa index of 0.91 (0.82-1.00).

| Scoring France | Scoring UK | | Total |
| --- | --- | --- | --- |
|  | Negative/Weak | Strong |  |
| Negative/weak | 33 | 3 | 36 |
| Strong | 1 | 57 | 58 |
| Total | 34 | 60 | 94 |

Multiplex ligation-dependent probe amplification (MLPA)

Genomic DNA from FFPE tumor tissue was extracted using the QIAamp DNA Mini Tissue Kit (Qiagen GmbH, Düsseldorf, Germany) according to the manufacturer’s instructions. Histological assessment of tissue fragments chosen for this study confirmed that all specimens consisted of at least 80% tumor cells.

For MLPA analysis, the SALSA MLPA SALSA MLPA p303-A1 (MRC-Holland, Amsterdam, Netherlands) assay was used. MLPA was performed in accordance with the manufacturer’s instructions. In brief, 100 ng DNA was denatured for 5 minutes and cooled down to 25°C. Following the addition of the probe mix, the sample was hybridized 16 hours at 60°C. After ligation, PCR was performed in a total volume of 50 µl containing 10 µl of the ligation mix on a thermocycler (Biometra, Jena, Germany). Subsequently, a LIZ-labelled internal size standard was added to the tumor samples and after denaturation fragments were separated and quantified on an ABI 3730 capillary sequencer (Applied Biosystems, Foster City, USA) and analysed using the Gene Mapper software (Applied Biosystems). Differences of +/- 3-fold SD from the mean were considered as significant gains or losses, respectively, after normalization of the assay using FFPE cerebellar tissue. As negative control, DNA from ependymomas that showed no gain of chromosome arm 1q using other techniques (CGH) was used. As positive controls we used DNA from ependymomas with confirmed gain of chromosome 1q by alternative methods (CGH, MIP, FISH).

**B: Details of the statistical analyses**

We used a sophisticated statistical analysis in order to capitalize the advantage of this unique large series of pediatric ependymomas. Considering that this population includes various strategies for the initial adjuvant treatment, i.e. chemotherapy only, radiotherapy only or both, we could compare the outcome of the patients according to the risk factors established at diagnosis. Since the treatment was the same (and still is the same in the current ongoing SIOPE and COG protocols) whatever the location of the tumor, we decided to analyses the risk factors on the whole cohort to increase the power to detect potential interactions between risk factors that may be otherwise missed in smaller cohorts where these adjustments may not be possible.

We detail below the different statistical methods used when constructing this prognostic score also called prognostic index. Different steps are needed to develop a prognostic score (Steyerberg EW). We have detailed them through the pooled analysis we conducted.

The first step consists in constructing a prognostic model using a multivariable Cox regression model stratified by cohort. The core model (model 1) included the standard clinicopathological variables potentially linked with prognosis in the literature (age, tumor location, grade, extent of resection and treatment). The two biomarkers, TNC and 1q gain were chosen from an extensive literature search and retested in at least two national cohorts. Only these two biomarkers proved to be statistically significant in at least two national cohorts (data not shown). We evaluated the added value of TNC (model 2); 1q25 gain (model 3); and the two markers at the same time (model 4) to the core model according to the two following statistical criteria: AIC (Akaike criterion) and iAUC (integrated Area Under Curve) measuring the quality of fit and discriminant ability, respectively (See Table D in S4 File, model 1-4).

The second step was to check the hypotheses underlying the selected Cox regression model developed in the first step (Harrell FE). As no continuous covariate was included in the model only the proportional hazard assumption of the Cox model had to be checked. This hypothesis assumes that the association between a predictor and overall survival is constant over-time. There are different approaches to test for a deviation from this hypothesis (Schöenfeld residual, interaction with time). In the paper, we used the Schöenfeld residual approach and concluded that this hypothesis was violated for radiotherapy (RT). Different approaches can be used to take it into account. We opted to the approach, which consists to use the upfront RT as a stratification variable. This approach provides no estimation of the association between upfront RT and the endpoint. Other approaches that consist in modeling the association between RT and overall survival as a time-dependent effect (either by a time by RT interaction or spline functions) are more difficult to interpret for clinicians and complicate the construction of a prognostic score. After a preliminary model was selected, some pre-specified interactions were tested one after the other and included in the model if statistically significant. This leads to define the final model. A resampling technique (bootstrap) was then used to evaluate the robustness of the final model (Sauerbrei). This was performed by testing if the markers of interest remained statistically significant when we randomly resampled with replacement of the original dataset. For each bootstrap sample we computed the frequency at which the covariate is statistically significant. The markers 1q25 gain, TNC and interaction between TNC and tumor location have a high frequency of selection (See footnote in Table E in S4 File).

The third step was to build a prognostic score based on the final Cox model. This score was derived from the linear predictor defined by the sum of the covariates included in the final model weighted by their estimated regression coefficients (See Table 1 for the score definition and Figure 2A for the histogram). It is useful to report the histogram of the prognostic score because its standard deviation is related to the discrimination ability (Royston): the larger the standard deviation, the higher the discrimination ability. The risk groups were derived by categorizing the prognostic score according to cut-points (See Table 1). The cut-points were defined according to a data-oriented method. The number of risk groups and the choice of cut-points are discussed by Royston: it is recommended to define 3 to 4 groups and not to use cut-points according to the endpoint (outcome-oriented approach). We chose to create 3 risk groups and defined cut-points using Cox’s method (27th and 73th percentiles) (Cox), even if other choices such as 25th and 75th percentiles have been used in the literature. Kaplan-Meier overall survival of the 3 risk groups was reported in Figure 2B and we estimated the hazard ratios of the risk group factors (defined as poor, intermediate and good) from a Cox model hence assessing the discrimination ability of the prognostic score. The model performance was also evaluated by the calibration of the model, i.e. the difference between observed and predicted survival probabilities (See Figure 2C).

The association between RT and overall survival was estimated in each risk group, separately. Since the design of the study was not randomized by RT, the association was estimated through a multivariable Cox model stratified on cohort and included measured confounders affecting both the decision to administrate upfront RT and overall survival (for example, age). However, we have to be cautious with the interpretation since it may be possible, in such observational study, that there are unmeasured confounders affecting both the decision to administrate upfront RT and overall survival. Considering that four out of five of the cohorts were composed of patients included in protocols, we may however assume that the impact of such factors was kept as low as possible.

The fourth step was to validate the prognostic score. We performed an internal validation using a cross validation (Royston) and not an external validation because there was no independent external data available to us. The general principle is to use patients from 4 cohorts out of 5, apply the final model defined in the previous step, build a prognostic score with the new estimation of the regression coefficients, define cut-points and risk groups from this score (step1). From the regression coefficients estimated from the 4 cohorts, we computed a prognostic score for each patient from the omitted cohort (this cohort is independent to the 4 other cohorts used to fit the final model) and assigned to each patient a risk group using the cut-points estimated in step 1 (step2). We repeated 5 times step1 and step 2 omitting at each time one different cohort. This means that each patient has been assigned to a risk group based on a score developed on an independent dataset. The last step (step 3) consists to estimate the Kaplan-Meier overall survival according to the 3 risk groups and to check whether the discrimination between the 3 risk groups is well retained (Figure 2D).

References:

Steyerberg EW. Clinical prediction models. A practical approach to development, validation, and updating. Springer editors, 2009

Harrell F, Lee KL, Mark DB. Tutorial in biostatistics. Multivariable prognostic models: issues in developing models, evaluating assumptions and adequacy, and measuring and reducing errors. Statistics in Medicine, 15, 361-387, 1996

Sauerbrei W, Schumacher M. A bootstrap resampling procedure for model building: Application to the Cox regression model. Statistics in Medicine, 11, 2093:2109, 1992

Royston P, Altman DG. External validation of a Cox prognostic model: principles and methods. BMC medical research methodology 2013, 13:33

Cox DR. Note on Grouping. Journal of the American statistical association, 52, n°280, 1957

Royston P, Parmar MKB, Sylvester R. Construction and validation of a prognostic model across several studies, with an application in superficial bladder cancer. Statistics in Medicine. ;23(6):907-926, 2004
