# Supplementary material for: Integrating Tenascin-C protein expression and 1q25 copy number status in pediatric intracranial ependymoma prognostication: A new model for risk stratification
Source: PLoS One. 2017 Jun 15;12(6):e0178351. doi: 10.1371/journal.pone.0178351 (PMC5472261; doi:10.1371/journal.pone.0178351)
Supplement: S2 File — (DOCX) [file pone.0178351.s002.docx]

**English Translation of the Informed Consent Form**

Informed Consent Form

for children below the age of 18 for the use of serum, plasma, cells or tissues, including tumor tissues obtained during the treatment in order to conduct scientific research

Madam, Sir,

Your child is followed by Dr ……………….. consulting at the Assistance Publique – Hôpitaux de Paris.

During the management of the disease of your child, the doctors will be conducted to take blood, cells or tissues that will be stored at the Centre de Ressources Biologiques de l’hôpital Necker Enfants Malades. These samples are taken to establish the diagnosis of the disease, the management and the follow-up of the treatment which could include a transplantation of cells. These samples will be used in the interest of your child to optimize the treatment.

To insure the quality of the treatment of your child, these samples will be conserved and frozen. This technique is very effective and allows to store them for years in good and safe conditions. The structure responsible for this mission is the Centre de Ressources Biologiques de l’hôpital Necker Enfants Malades – Assistance Publique – Hôpitaux de Paris: it is the tumor bank.

Part of the samples of your child may not be necessary for the diagnosis and treatment. These remaining samples represent a great resource for the conduct of scientific research. We would like to obtain your agreement to perform scientific research with the remaining samples. These research projects will not have any consequences on the treatment of your child.

If the use of the samples may comprise a study of genetic characteristics of your child, ie constitutional genomics, you will be informed of the nature and objectives of this study. Your consent and the one from your child if able to give it will be requested to perform these analyses.

The research programmes developed at Necker Enfants Malades – Assistance Publique – Hôpitaux de Paris are aiming at discovering the mechansims of diseases or new therapeutics. These programmes could be organized in the frame of the Programme Hospitalier de Recherche Clinique funded by the Ministry of Health, or alternative research programmes supported by the Assistance Publique – Hôpitaux de Paris, the Institut National de la Santé et de la Recherche Médicale (INSERM), the Centre National de la Recherche Scientifique (CNRS) or collaborations with the pharmaceutical industries.

The results of these research projects may be published in scientific journals. Knwoledge and innovations of these researches may also lead to patent applications. These patents could be sold to third parties such as pharmaceutical industries interested in the development of diagnostic tests or new treatments for the children. In case these research projects will produce financial benefits for the Assistance Publique – Hôpitaux de Paris, which is a public organization, they would be reinvested as mentioned in the law for intellectual property in the research programmes developed by the Assistance Publique – Hôpitaux de Paris. As indicated in the European Directive 98/44/CE an application for a patent can only be made with the approval of the donor or the guardians.

For all these reasons, we would like to obtain your consent, and the one from your child if able too give it, to conduct these research projects with the above mentioned samples. You will sign and date this document and return it to the above mentioned doctor.

Of course, you and your child have the right to withdrawn your consent anytime, without the necessity to explain why. You would just have to inform us.

If you do no sign this inform consent form, the samples taken from your child will not be used for research purposes.

As for every research on patient’s material, we may need to use clinical information. As you know, these informations are recorded in the clinical files. The Commission Nationale de l’Informatique et des Libertés has allow the automatic and anonymous analyses of these clinical data.

As part of the samples from your child may be extremely valuable for the scientific research, clinical data in confrontation with the results of the biological research projects will produce new and important knowledge about diseases and their management.

You or your child may decide to refuse the use of clinical data for the research. In this case, none of the clinical information will be used for the research.

Once you would have signed this document, your approval for these researches and the anonymous use of the clinical data will be notified in the hospital files of your child.

Madam, Sir,

Whatever your decision will be, we would like to thank you for your attention given to read this document.

If you think that the information you received was insufficient or if you would need additional information, you can contact the physician treating your child or directly writing to:

Centre de Ressources Biologiques

Hôpital necker Enfants Malades

Assistance Publique – Hôpitaux de Paris

149 rue de Sèvres 75743 Paris cedex 15

**Consent**

We have read and understood the document indicating that the Centre de Ressources Biologiques de l’hôpital Necker Enfants Malades – Assistance Publique – Hôpitaux de Paris has the mission to store the serum, the plasma, the cells, the tissues, including the tumor tissues collected in the interest of my child to allow the correct management of his disease but that part of the samples not used for the diagnosis, the treatment and the follow-up may be used for scientific researches.

We know that they could be used by researchers working at the Assistance Publique – Hôpitaux de Paris or by researchers working outside this institution in the frame of specific research programmes.

We know that the clinical data would be matched anonymously with the biological results.

We consider that we have been informed appropriately.

We give our approval for the use of the remaining samples for research purpose.

We do not give our approval for the use of the remaining samples for research purpose.

We know that our decision can be changed anytime and this the consent could be withdrawn.

Signatures.
