# Supplementary figures and images for: Integrating Tenascin-C protein expression and 1q25 copy number status in pediatric intracranial ependymoma prognostication: A new model for risk stratification
Source: PLoS One. 2017 Jun 15;12(6):e0178351. doi: 10.1371/journal.pone.0178351 (PMC5472261; doi:10.1371/journal.pone.0178351)

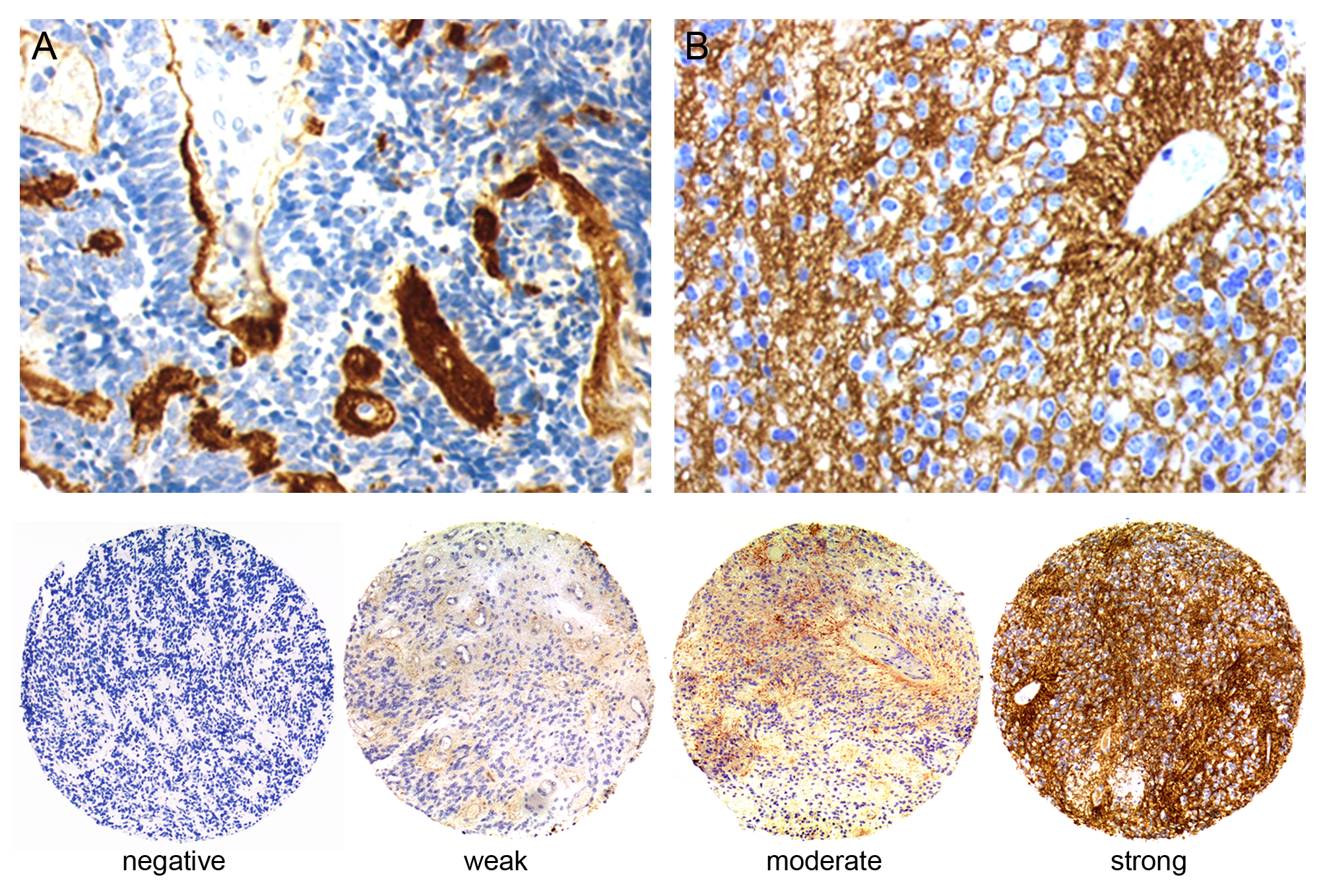

Supplement: S3 File — —Fig A. TNC immunostaining in pediatric ependymoma. Upper panel: qualitative aspects of TNC staining: (A) Perivascular staining; (B) Perivascular and intercellular staining. Lower panel: TNC scoring: most positive areas were analyzed and scored for intensity of staining as shown. Only moderate and strong staining were considered as overexpression; Fig B. Flow chart; Fig C. Kaplan-Meier-based overall survival curves overall (dashed lines represent the 95% confidence bands) and by cohort (n = 478); Fig D. Kaplan-Meier-based overall survival by radiotherapy, for good (A), intermediate (B) and poor (C) risk groups. (ZIP) [file pone.0178351.s003.zip › Fig A.tif]

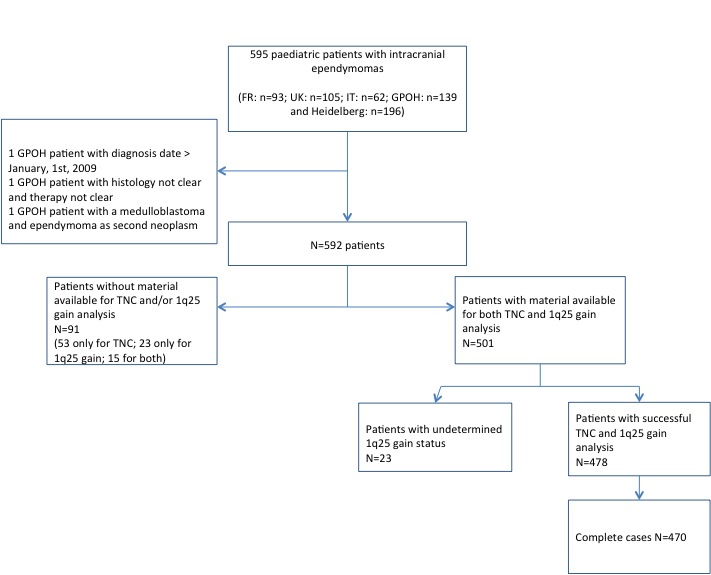

Supplement: S3 File — —Fig A. TNC immunostaining in pediatric ependymoma. Upper panel: qualitative aspects of TNC staining: (A) Perivascular staining; (B) Perivascular and intercellular staining. Lower panel: TNC scoring: most positive areas were analyzed and scored for intensity of staining as shown. Only moderate and strong staining were considered as overexpression; Fig B. Flow chart; Fig C. Kaplan-Meier-based overall survival curves overall (dashed lines represent the 95% confidence bands) and by cohort (n = 478); Fig D. Kaplan-Meier-based overall survival by radiotherapy, for good (A), intermediate (B) and poor (C) risk groups. (ZIP) [file pone.0178351.s003.zip › Fig B.tif]

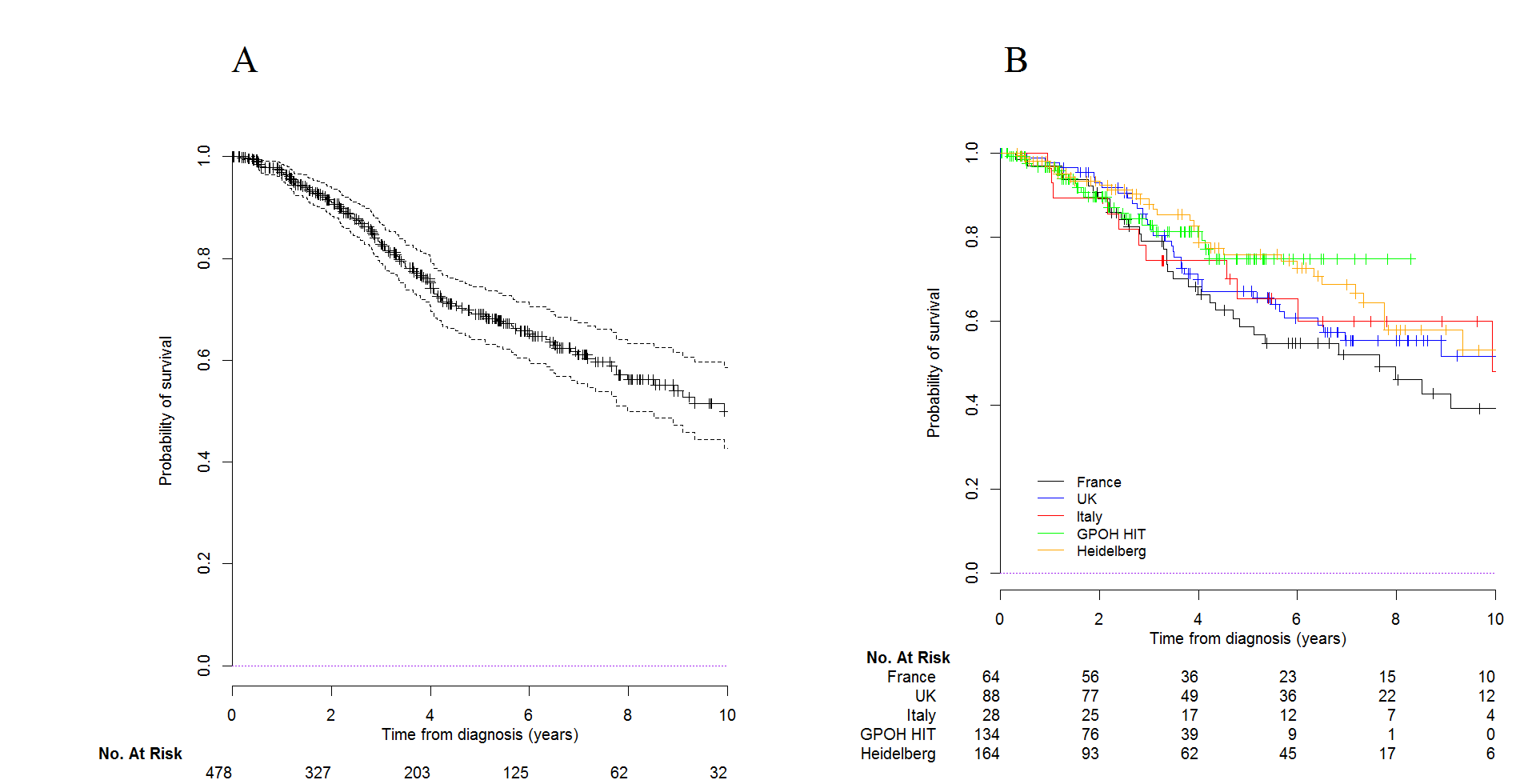

Supplement: S3 File — —Fig A. TNC immunostaining in pediatric ependymoma. Upper panel: qualitative aspects of TNC staining: (A) Perivascular staining; (B) Perivascular and intercellular staining. Lower panel: TNC scoring: most positive areas were analyzed and scored for intensity of staining as shown. Only moderate and strong staining were considered as overexpression; Fig B. Flow chart; Fig C. Kaplan-Meier-based overall survival curves overall (dashed lines represent the 95% confidence bands) and by cohort (n = 478); Fig D. Kaplan-Meier-based overall survival by radiotherapy, for good (A), intermediate (B) and poor (C) risk groups. (ZIP) [file pone.0178351.s003.zip › Fig C.tif]

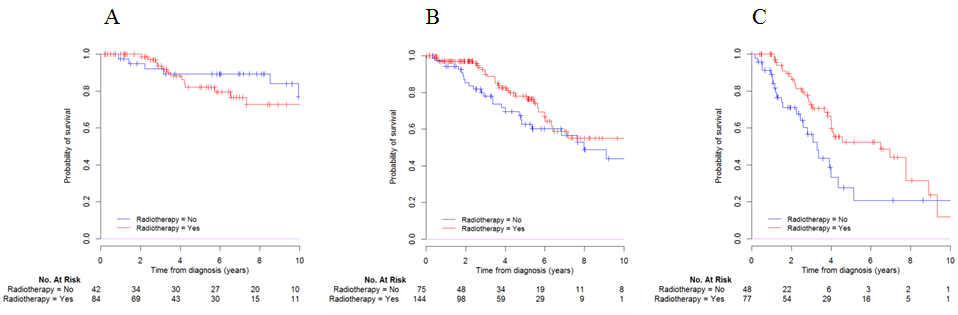

Supplement: S3 File — —Fig A. TNC immunostaining in pediatric ependymoma. Upper panel: qualitative aspects of TNC staining: (A) Perivascular staining; (B) Perivascular and intercellular staining. Lower panel: TNC scoring: most positive areas were analyzed and scored for intensity of staining as shown. Only moderate and strong staining were considered as overexpression; Fig B. Flow chart; Fig C. Kaplan-Meier-based overall survival curves overall (dashed lines represent the 95% confidence bands) and by cohort (n = 478); Fig D. Kaplan-Meier-based overall survival by radiotherapy, for good (A), intermediate (B) and poor (C) risk groups. (ZIP) [file pone.0178351.s003.zip › Fig D.tif]
