# Supplementary material for: Integrating Tenascin-C protein expression and 1q25 copy number status in pediatric intracranial ependymoma prognostication: A new model for risk stratification
Source: PLoS One. 2017 Jun 15;12(6):e0178351. doi: 10.1371/journal.pone.0178351 (PMC5472261; doi:10.1371/journal.pone.0178351)
Supplement: S4 File — —Table A. Baseline characteristics, by cohort and for all patients; Table B. Patient and tumor characteristics for patients with and without TNC and 1q25 gain results; Table C. Correlation between Tenascin-C and 1q25 gain and baseline characteristics in all patients—complete cases analysis; Table D. Analysis of overall survival (OS) using a multivariable Cox regression model stratified by cohort in complete cases; Table E. Analysis of overall survival (OS) using a multivariable Cox regression model without and with interaction between TNC and tumor location stratified by cohort and radiotherapy in complete cases; Table F. P-values of pre-specified interaction terms; Table G. Baseline characteristics, by cohort and overall in posterior fossa patients; Table H. Baseline characteristics, by cohort and overall in supratentorial patients. (ZIP) [file pone.0178351.s004.zip › Table A.docx]

Table A: Baseline characteristics, by cohort and for all patients (n=478)^†^

| Characteristics | France  (n=64) | UK  (n=88) | Italy  (n=28) | GPOH HIT  (n=134) | Heidelberg  (n=164) | Total  (n=478) | p value* |
| --- | --- | --- | --- | --- | --- | --- | --- |
|  | N (%) | N (%) | N (%) | N (%) | N (%) | N (%) |  |
| Sex  Male  Female | 34 (53)  30 (47) | 55 (62)  33 (38) | 16 (57)  12 (43) | 81 (60)  53 (40) | 105 (64)  59 (36) | 291 (61)  187 (39) | 0.63 |
| Age at diagnosis (months)  median [Min - Max] | 32 [7; 166] | 31 [4; 200] | 35 [4; 173] | 50 [3; 223] | 72 [12; 216] | 48 [3; 223] | <0.0001 |
| Age at diagnosis  <36months  ≥ 36 months | 35 (55)  29 (45) | 50 (57)  38 (43) | 15 (54)  13 (46) | 48 (36)  86 (64) | 28 (17)  136 (83) | 176 (37)  302 (63) | <0.0001 |
| Tumor location  Posterior fossa  Supratentorial | 47 (73)  17 (27) | 66 (75)  22 (25) | 21 (75)  7 (25) | 86 (64)  48 (36) | 110 (67)  54 (33) | 330 (69)  148 (31) | 0.37 |
| Grade  II  III | 13 (20)  51 (80) | 49 (56)  39 (44) | 14 (50)  14 (50) | 23 (17)  111 (83) | 38 (23)  126 (77) | 137 (29)  341 (71) | <0.0001 |
| Extent of resection  Incomplete  Complete  Missing | 24 (38)  40 (62) | 46 (52)  42 (48) | 8 (29)  20 (71) | 48 (38)  80 (62)  6 | 86 (52)  78 (48) | 212 (45)  260 (55)  6 | 0.011 |
| Radiotherapy^¥^  No  Yes  Missing | 45 (70)  19 (30) | 29 (33)  59 (67) | 15 (54)  13 (46) | 13 (10)  119 (90)  2 | 65 (40)  99 (60) | 167 (35)  309 (65)  2 | <0.0001 |
|  | | | | | | |  |
| Tenascin-C  Negative  Positive | 24 (38)  40 (62) | 36 (41)  52 (59) | 10 (36)  18 (64) | 56 (42)  78 (58) | 80 (49)  84 (51) | 206 (43)  272 (57) | 0.42 |
| 1q25 gain  Negative  Positive | 50 (78)  14 (22) | 70 (80)  18 (20) | 24 (86)  4 (14) | 109 (81)  25 (19) | 134 (82)  30 (18) | 387 (81)  91 (19) | 0.92 |
| RELA  Negative  Positive  Missing | 53 (83)  11 (17) | 53 (90)  6 (10)  29 | 1 (20)  4 (80)  23 | 0 (0)  0 (0)  134 | 96 (73)  35 (27)  33 | 203 (78)  56 (22)  219 | <0.0001 |
|  | | | | | | |  |
| Median follow-up  [range] (years) | 8.0  [1.9; 16.9] | 7.1  [0.0; 15.2] | 7.5  [2.1; 12.5] | 2.9  [0.0; 8.3] | 3.0  [0.3; 17.0] | 5.0  [0.0; 17.0] | <0.0001 |
| Number of death | 33 (52) | 34 (39) | 12 (43) | 20 (15) | 33 (20) | 132 (28) | <0.0001 |
| Overall survival  median [95%CI] (years) | 7.66  [4.70; NA] | NA  [6.41; NA] | 9.94  [4.79; NA] | NA  [NA; NA] | 11.00  [7.75; NA] | 9.94  [7.98; NA] | 0.26^£^ |
| Number of events | 47 (73) | 51 (58) | 19 (68) | 40 (30) | 70 (43) | 227 (47) | <0.0001 |
| Event free survival  median [95%CI]  (years) | 1.9  [1.1; 3.5] | 2.8  [2.2; 5.7] | 1.6  [0.8; 3.6] | 5.6  [5.6; NA] | 5.3  [2.4; 7.7] | 3.8  [2.6; 5.6] | <0.001^£^ |

^†^: Patients with both TNC and 1q25 gain results; ^¥^: As no detailed information about treatment exist in Heidelberg cohort, we standardized treatment (None, chemotherapy alone, chemotherapy + radiotherapy and radiotherapy alone) in the other cohorts as radiotherapy (no, yes) in order to combine all cohorts. NA: Not assessable; *: p-values were estimated using the Chi2 or Fisher exact, Kruskal-Wallis and logrank tests for binary, continuous and censored data, respectively; ^£^ : indicates the p-value comparing the whole curve across country
