# Supplementary material for: Integrating Tenascin-C protein expression and 1q25 copy number status in pediatric intracranial ependymoma prognostication: A new model for risk stratification
Source: PLoS One. 2017 Jun 15;12(6):e0178351. doi: 10.1371/journal.pone.0178351 (PMC5472261; doi:10.1371/journal.pone.0178351)
Supplement: S4 File — —Table A. Baseline characteristics, by cohort and for all patients; Table B. Patient and tumor characteristics for patients with and without TNC and 1q25 gain results; Table C. Correlation between Tenascin-C and 1q25 gain and baseline characteristics in all patients—complete cases analysis; Table D. Analysis of overall survival (OS) using a multivariable Cox regression model stratified by cohort in complete cases; Table E. Analysis of overall survival (OS) using a multivariable Cox regression model without and with interaction between TNC and tumor location stratified by cohort and radiotherapy in complete cases; Table F. P-values of pre-specified interaction terms; Table G. Baseline characteristics, by cohort and overall in posterior fossa patients; Table H. Baseline characteristics, by cohort and overall in supratentorial patients. (ZIP) [file pone.0178351.s004.zip › Table B.docx]

Table B: Patient and tumor characteristics for patients with and without TNC and 1q25 gain results (N=478)

| Characteristics | Patients with TNC status unknown and/or 1q25 gain status unknown*  (N=114) | Patients with both TNC and 1q25 gain status known  (N=478) | p-values† |
| --- | --- | --- | --- |
|  | N (%) | N (%) |  |
| Cohort  FR  UK  IT  GPOH HIT  Heidelberg | 29 (25)  17 (15)  34 (30)  2 (2)  32 (28) | 64 (13)  88 (18)  28 (6)  134 (28)  164 (34) | <0.0001‡ |
| Sex  Male  Female | 63 (55)  51 (45) | 291 (61)  187 (39) | 0.4394 |
| Age at diagnosis  <36months  ≥ 36 months | 44 (39)  70 (61) | 176 (37)  302 (63) | 0.5115 |
| Tumor location  Posterior fossa  Supratentorial | 74 (65)  40 (35) | 330 (69)  148 (31) | 0.2298 |
| Grade  II  III | 37 (32)  77 (68) | 137 (29)  341 (71) | 0.5305 |
| Extent of resection  Incomplete  Complete  Missing | 43 (38)  71 (62)  0 | 212 (45)  260 (55)  6 | 0.4140 |
| Radiotherapy  No  Yes  Missing | 50 (44)  64 (56)  0 | 167 (35)  309 (65)  2 | 0.4038 |

*: Patients without TNC status and/or without 1q25 gain status include (i) patients without tumor tissue samples (n=91: 53 only for TNC, 23 only for 1q25 gain and 15 for both markers) and (ii) patients without results after TNC and 1q25 gain analyses (n=23 for 1q25 gain); †: Cochran-Mantel-Haenszel test after stratifying for cohorts (missing data were excluded); ‡: Chi2 test
