# Supplementary material for: Integrating Tenascin-C protein expression and 1q25 copy number status in pediatric intracranial ependymoma prognostication: A new model for risk stratification
Source: PLoS One. 2017 Jun 15;12(6):e0178351. doi: 10.1371/journal.pone.0178351 (PMC5472261; doi:10.1371/journal.pone.0178351)
Supplement: S4 File — —Table A. Baseline characteristics, by cohort and for all patients; Table B. Patient and tumor characteristics for patients with and without TNC and 1q25 gain results; Table C. Correlation between Tenascin-C and 1q25 gain and baseline characteristics in all patients—complete cases analysis; Table D. Analysis of overall survival (OS) using a multivariable Cox regression model stratified by cohort in complete cases; Table E. Analysis of overall survival (OS) using a multivariable Cox regression model without and with interaction between TNC and tumor location stratified by cohort and radiotherapy in complete cases; Table F. P-values of pre-specified interaction terms; Table G. Baseline characteristics, by cohort and overall in posterior fossa patients; Table H. Baseline characteristics, by cohort and overall in supratentorial patients. (ZIP) [file pone.0178351.s004.zip › Table C.docx]

Table C: Correlation between Tenascin-C and 1q25 gain and baseline characteristics in all patients – complete cases analysis (n=470)

| Prognostic factor | Tenascin-C | | | | 1q25 gain | | | |
| --- | --- | --- | --- | --- | --- | --- | --- | --- |
|  | Negative (n=203) | Positive (n=267) | p-value^†^ | Negative (n=382) | | Positive (n=88) | p-value^†^ |  |
|  | n (%) | n (%) |  | n (%) | | n (%) |  |  |
| Sex  Male  Female | 122 (42)  81 (45) | 166 (58)  101 (55) | 0.5665 | 228 (79)  154 (85) | | 60 (21)  28 (15) | 0.1330 |  |
| Age at diagnosis  <36months  ≥ 36 months | 41 (24)  162 (55) | 132 (76)  135 (45) | <0.0001¶ | 150 (87)  232 (78) | | 23 (13)  65 (22) | 0.0095 ¶ |  |
| Tumor location Posterior fossa  Supratentorial | 101 (31)  102 (70) | 224 (69)  43 (30) | <0.0001¶ | 256 (79)  126 (87) | | 69 (21)  19 (13) | 0.0413¶ |  |
| Grade  II  III | 59 (43)  144 (43) | 78 (57)  189 (57) | 0.8511 | 111 (81)  271 (81) | | 26 (19)  62 (19) | 0.9637 |  |
| Extent of resection  Incomplete  Complete | 82 (39)  121 (47) | 129 (61)  138 (53) | 0.0487 | 173 (82)  209 (81) | | 38 (18)  50 (19) | 0.6764 |  |
| Radiotherapy  No  Yes | 60 (36)  143 (47) | 105 (64)  162 (53) | 0.0259 | 138 (84)  244 (80) | | 27 (16)  61 (20) | 0.2024 |  |
| 1q25 gain  Negative  Positive | 164 (43)  39 (44) | 218 (57)  49 (56) | 0.7984 |  | |  |  |  |
| RELA  Negative  Positive | 79 (39)  29 (52) | 124 (61)  27 (48) | 0.1589 | 161 (79)  46 (82) | | 42 (21)  10 (18) | 0.8232 |  |

^†^: p-value was computed using the Cochran-Mantel-Haenszel test after stratifying for cohorts, ¶: indicate that these associations remain statistically significant after adjustment on all covariates (except 1q25 gain) using a logistic regression stratified by cohort (we did not include RELA because of large missing data)
