# Supplementary material for: Integrating Tenascin-C protein expression and 1q25 copy number status in pediatric intracranial ependymoma prognostication: A new model for risk stratification
Source: PLoS One. 2017 Jun 15;12(6):e0178351. doi: 10.1371/journal.pone.0178351 (PMC5472261; doi:10.1371/journal.pone.0178351)
Supplement: S4 File — —Table A. Baseline characteristics, by cohort and for all patients; Table B. Patient and tumor characteristics for patients with and without TNC and 1q25 gain results; Table C. Correlation between Tenascin-C and 1q25 gain and baseline characteristics in all patients—complete cases analysis; Table D. Analysis of overall survival (OS) using a multivariable Cox regression model stratified by cohort in complete cases; Table E. Analysis of overall survival (OS) using a multivariable Cox regression model without and with interaction between TNC and tumor location stratified by cohort and radiotherapy in complete cases; Table F. P-values of pre-specified interaction terms; Table G. Baseline characteristics, by cohort and overall in posterior fossa patients; Table H. Baseline characteristics, by cohort and overall in supratentorial patients. (ZIP) [file pone.0178351.s004.zip › Table D.docx]

Table D: Analysis of overall survival (OS) using a multivariable Cox regression model stratified by cohort in complete cases (n=470)

| Prognostic factor |  | Multivariable | | | |
| --- | --- | --- | --- | --- | --- |
|  |  | Model 1 | Model 2 | Model 3 | Model 4 |
|  |  | Model 1  (core model) | Model 1  + Tenascin-C | Model 1  + 1q25 gain | Model 1  + Tenascin-C + 1q25 gain |
|  | No. patients / deaths | HR [95%CI]  (p-value) ^†^ | HR [95%CI]  (p-value) ^†^ | HR [95%CI]  (p-value) ^†^ | HR [95%CI]  (p-value) ^†^ |
| Age at diagnosis  <36months  ≥ 36 months | 173/63  297/68 | 1.0  0.829 [0.525; 1.310]  (0.4215) | 1.0  0.914 [0.577; 1.447]  (0.7002) | 1.0  0.713 [0.453; 1.124]  (0.1453) | 1.0  0.796 [0.502; 1.260]  (0.3301) |
| Tumor location  Posterior fossa  Supratentorial | 325/100  145/31 | 1.0  0.754 [0.495; 1.148]  (0.1873) | 1.0  0.864 [0.555; 1.343]  (0.5151) | 1.0  0.798 [0.523; 1.217]  (0.2945) | 1.0  0.892 [0.577; 1.379]  (0.6070) |
| Grade  II  III | 137/37  333/94 | 1.0  1.857 [1.204; 2.864]  (0.0051) | 1.0  1.841 [1.191; 2.846]  (0.0060) | 1.0  1.939 [1.252; 3.004]  (0.0030) | 1.0  1.966 [1.265; 3.056]  (0.0026) |
| Extent of resection  Incomplete  Complete | 211/67  259/64 | 1.0  0.536 [0.372; 0.772]  (0.0008) | 1.0  0.558 [0.386; 0.805]  (0.0018) | 1.0  0.492 [0.339; 0.714]  (0.0002) | 1.0  0.521 [0.358; 0.757]  (0.0006) |
| Radiotherapy  No  Yes | 165/55  305/76 | 1.0  0.930 [0.582; 1. 484]  (0.7603) | 1.0  0.904 [0.566; 1.442]  (0.6703) | 1.0  0.909 [0.569; 1.450]  (0.6883) | 1.0  0.872 [0.545; 1.395]  (0.5675) |
| Tenascin-C  Negative  Positive | 203/45  267/86 |  | 1.0  1.482 [0.992; 2.212]  (0.0546) |  | 1.0  1.487 [0.999; 2.215]  (0.0508) |
| 1q25 gain  Negative  Positive | 382/88  88/43 |  |  | 1.0  2.833 [1.927; 4.164]  (<0.0001) | 1.0  2.836 [1.930; 4.166]  (<0.0001) |
| Performance model^¶^  AIC  iAUC |  | 992.7654  0.631 | 990.9896  0.642 | 969.7215  0.695 | 967.8221  0.702 |

^†^: p-value was calculated from the Wald-test, ^¶^: AIC: Akaike measures the overall performance and iAUC: integral of AUC on time interval [0, T] with T=3 years measures the discriminant ability
