# Supplementary material for: Integrating Tenascin-C protein expression and 1q25 copy number status in pediatric intracranial ependymoma prognostication: A new model for risk stratification
Source: PLoS One. 2017 Jun 15;12(6):e0178351. doi: 10.1371/journal.pone.0178351 (PMC5472261; doi:10.1371/journal.pone.0178351)
Supplement: S4 File — —Table A. Baseline characteristics, by cohort and for all patients; Table B. Patient and tumor characteristics for patients with and without TNC and 1q25 gain results; Table C. Correlation between Tenascin-C and 1q25 gain and baseline characteristics in all patients—complete cases analysis; Table D. Analysis of overall survival (OS) using a multivariable Cox regression model stratified by cohort in complete cases; Table E. Analysis of overall survival (OS) using a multivariable Cox regression model without and with interaction between TNC and tumor location stratified by cohort and radiotherapy in complete cases; Table F. P-values of pre-specified interaction terms; Table G. Baseline characteristics, by cohort and overall in posterior fossa patients; Table H. Baseline characteristics, by cohort and overall in supratentorial patients. (ZIP) [file pone.0178351.s004.zip › Table E.docx]

Table E: Analysis of overall survival (OS) using a multivariable Cox regression model without and with interaction between TNC and tumor location stratified by cohort and radiotherapy in complete cases (n=470)

|  | Model without interaction | Model with interaction^†^ |
| --- | --- | --- |
| Prognostic factor | HR [95%CI] (p-value) | HR [95%CI] (p-value) |
| Age at diagnosis  <36months  ≥ 36 months | 1.0  0.760 [0.484; 1.194] (0.2335) | 1.0  0.916 [0.579; 1.447] (0.7058) |
| Tumor location  Posterior fossa  Supratentorial | 1.0  0.925 [0.603; 1.419] (0.7218) | 1.0  1.844 [0.981; 3.466] (0.0573) |
| Grade  II  III | 1.0  1.807 [1.170; 2.791] (0.0077) | 1.0  1.940 [1.245; 3.022] (0.0034) |
| Extent of resection  Incomplete  Complete | 1.0  0.527 [0.362; 0.768] (0.0008) | 1.0  0.560 [0.384; 0.818] (0.0028) |
| Tenascin-C*  Positive vs Negative |  | Posterior fossa: 2.197 [1.286; 3.755] (0.0040)  Supratentorial: 0.639 [0.277; 1.477] (0.2950) |
| 1q25 gain  Negative  Positive | 1.0  2.713 [1.834; 4.012] (<0.0001) | 1.0  2.969 [1.992; 4.425] (<0.0001) |
| Performance model^¶^  AIC  iAUC | 823.7734  0.677 | 817.4268  0.701 |

^†^: As an interaction between Tenascin-C and tumor location is identified, we represented the effect of TNC according to tumor location modalities.

*: AIC=821.9 and iAUC=0.687 for the model reported in the second column plus TNC (with HR of TNC=1.513 [0.996; 2.297] p=0.0519).

^¶^: AIC: Akaike measures the overall performance and iAUC: integral of AUC on time interval [0, T] with T=3 years measures the discriminant ability

We used a bootstrap resampling (B=1000) to evaluate the stability of the final model. This technique consists of fitting the model on B bootstrap samples and to estimate the frequency of a statistically significant association (alpha=0.05) for each covariate with OS. The percentages were 99% for 1q25 gain, 81% for TNC, 84% for extent of resection, 78% for grade, 68% for interaction between TNC and tumor location, 47% for tumor location and 7% for age.
