# Supplementary material for: Integrating Tenascin-C protein expression and 1q25 copy number status in pediatric intracranial ependymoma prognostication: A new model for risk stratification
Source: PLoS One. 2017 Jun 15;12(6):e0178351. doi: 10.1371/journal.pone.0178351 (PMC5472261; doi:10.1371/journal.pone.0178351)
Supplement: S4 File — —Table A. Baseline characteristics, by cohort and for all patients; Table B. Patient and tumor characteristics for patients with and without TNC and 1q25 gain results; Table C. Correlation between Tenascin-C and 1q25 gain and baseline characteristics in all patients—complete cases analysis; Table D. Analysis of overall survival (OS) using a multivariable Cox regression model stratified by cohort in complete cases; Table E. Analysis of overall survival (OS) using a multivariable Cox regression model without and with interaction between TNC and tumor location stratified by cohort and radiotherapy in complete cases; Table F. P-values of pre-specified interaction terms; Table G. Baseline characteristics, by cohort and overall in posterior fossa patients; Table H. Baseline characteristics, by cohort and overall in supratentorial patients. (ZIP) [file pone.0178351.s004.zip › Table F.docx]

Table F: P-values of pre-specified interaction terms.

| Prognostic factor | Prognostic factor | Overall Survival |
| --- | --- | --- |
|  |  | p-value†* |
| Age at diagnosis | Grade  Tenascin-C  1q25 gain | 0.052  0.250  0.274 |
| Tumor location | Grade  Extent of resection  Tenascin-C  1q25 gain | 0.218  0.379  0.014  0.058 |
| Grade | Tenascin-C  1q25 gain | 0.529  0.674 |
| Extent of resection | Tenascin-C | 0.110 |
| Tenascin-C | 1q25 gain  Radiotherapy  Cohort | 0.761  0.156  0.304 |
| 1q25 gain | Radiotherapy  Cohort | 0.893  0.158 |

†: p-value is computed when we added, one at a time, an interaction term in the multivariable Cox regression model (age at diagnosis + tumor location + grade + extent of resection + 1q25 gain) stratified by cohort and radiotherapy. However, as TNC was not initially selected, interaction between this marker and other covariates required the inclusion of both main effect and interaction term.
