# Supplementary material for: Integrating Tenascin-C protein expression and 1q25 copy number status in pediatric intracranial ependymoma prognostication: A new model for risk stratification
Source: PLoS One. 2017 Jun 15;12(6):e0178351. doi: 10.1371/journal.pone.0178351 (PMC5472261; doi:10.1371/journal.pone.0178351)
Supplement: S4 File — —Table A. Baseline characteristics, by cohort and for all patients; Table B. Patient and tumor characteristics for patients with and without TNC and 1q25 gain results; Table C. Correlation between Tenascin-C and 1q25 gain and baseline characteristics in all patients—complete cases analysis; Table D. Analysis of overall survival (OS) using a multivariable Cox regression model stratified by cohort in complete cases; Table E. Analysis of overall survival (OS) using a multivariable Cox regression model without and with interaction between TNC and tumor location stratified by cohort and radiotherapy in complete cases; Table F. P-values of pre-specified interaction terms; Table G. Baseline characteristics, by cohort and overall in posterior fossa patients; Table H. Baseline characteristics, by cohort and overall in supratentorial patients. (ZIP) [file pone.0178351.s004.zip › Table G.docx]

Table G: Baseline characteristics, by cohort and overall in posterior fossa patients (n=330)^†^

| Characteristics | France  (n=47) | UK  (n=66) | Italy  (n=21) | GPOH HIT  (n=86) | Heidelberg  (n=110) | Total  (n=330) | p value* |
| --- | --- | --- | --- | --- | --- | --- | --- |
|  | N (%) | N (%) | N (%) | N (%) | N (%) | N (%) |  |
| Sex  Male  Female | 26 (55)  21 (45) | 46 (70)  20 (30) | 14 (67)  7 (33) | 51 (59)  35 (41) | 79 (72)  31 (28) | 216 (65)  114 (35) | 0.19 |
| Age at diagnosis (months)  median [Min - Max] | 28 [7-164] | 30 [9-200] | 35 [4-159] | 45 [5-190] | 72 [12-216] | 40 [4; 216] | <0.0001 |
| Age at diagnosis  <36months  ≥ 36 months | 29 (62)  18 (38) | 43 (65)  23 (35) | 12 (57)  9 (43) | 36 (42)  50 (58) | 18 (16)  92 (84) | 138 (42)  192 (58) | <0.0001 |
| Grade  II  III  Missing | 10 (21)  37 (79) | 40 (61)  26 (39) | 12 (57)  9 (43) | 20 (23)  66 (77) | 31 (28)  79 (72) | 113 (34)  217 (66) | <0.0001 |
| Extent of resection  Incomplete  Complete  Missing | 15 (32)  32 (68) | 36 (55)  30 (45) | 8 (38)  13 (62) | 32 (39)  50 (61)  4 | 61 (55)  49 (45) | 152 (47)  174 (53)  4 | 0.02 |
| Radiotherapy^¥^  No  Yes  Missing | 36 (77)  11 (23) | 22 (33)  44 (67) | 12 (57)  9 (43) | 11 (13)  74 (87)  1 | 47 (43)  63 (57) | 128 (39)  201 (61)  1 | <0.0001 |
|  | | | | | | |  |
| Tenascin-C  Negative  Positive | 11 (23)  36 (77) | 18 (27)  48 (73) | 6 (29)  15 (71) | 17 (20)  69 (80) | 50 (45)  60 (55) | 102 (31)  228 (69) | 0.001 |
| 1q25 gain  Negative  Positive | 37 (79)  10 (21) | 51 (77)  15 (23) | 17 (81)  4 (19) | 68 (79)  18 (21) | 86 (78)  24 (22) | 259 (78)  71 (22) | 0.99 |
| RELA  Negative  Positive  Missing | 47 (100)  0 (0) | 46 (94)  3 (6)  17 | 0 (0)  0 (0)  21 | 0 (0)  0 (0)  86 | 91 (100)  0 (0)  19 | 184 (98)  3 (2)  143 | <0.0001 |
|  | | | | | | |  |
| Median follow-up  [range] (years) | 8.7  [2.0; 15.3] | 8.2  [0.0; 15.2] | 7.5  [2.1; 11.5] | 2.9  [0.2; 8.3] | 3.9  [0.3; 17.0] | 5.4  [0.0; 17.0] | <0.0001 |
| Number of death | 28 (60) | 30 (45) | 9 (43) | 15 (17) | 19 (17) | 101 (31) | <0.0001 |
| Overall survival  median [95%CI] (years) | 6.8  [4.3 ; 10.6] | 7.0  [5.5 ; NA] | 9.9  [4.6 ; NA] | NA  [NA ; NA] | NA  [9.3 ; NA] | 9.3  [7.7 ; NA] | 0.06^£^ |
| Number of events | 36 (77) | 41 (62) | 15 (71) | 31 (36) | 46 (42) | 169 (51) | <0.0001 |
| Event free survival  median [95%CI] (years) | 1.9  [1.1 ; 3.9] | 2.6  [2.2 ; 5.7] | 0.9  [0.7 ; NA] | 5.6  [2.5 ; NA] | 6.0  [3.1 ; NA] | 3.4  [2.3 ; 5.3] | 0.003^£^ |

^†^: Patients with both TNC and 1q25 gain results; ^¥^: As no detailed information about treatment exist in Heidelberg cohort, we standardized treatment (None, chemotherapy alone, chemotherapy + radiotherapy and radiotherapy alone) in the other cohorts as radiotherapy (no, yes) in order to combine all cohorts. NA: Not assessable; *: p-values were estimated using the Chi2, Kruskal-Wallis and logrank tests for binary, continuous and censored data, respectively; ^£^ : indicates the p-value comparing the whole curve across country
