# Supplementary material for: Integrating Tenascin-C protein expression and 1q25 copy number status in pediatric intracranial ependymoma prognostication: A new model for risk stratification
Source: PLoS One. 2017 Jun 15;12(6):e0178351. doi: 10.1371/journal.pone.0178351 (PMC5472261; doi:10.1371/journal.pone.0178351)
Supplement: S4 File — —Table A. Baseline characteristics, by cohort and for all patients; Table B. Patient and tumor characteristics for patients with and without TNC and 1q25 gain results; Table C. Correlation between Tenascin-C and 1q25 gain and baseline characteristics in all patients—complete cases analysis; Table D. Analysis of overall survival (OS) using a multivariable Cox regression model stratified by cohort in complete cases; Table E. Analysis of overall survival (OS) using a multivariable Cox regression model without and with interaction between TNC and tumor location stratified by cohort and radiotherapy in complete cases; Table F. P-values of pre-specified interaction terms; Table G. Baseline characteristics, by cohort and overall in posterior fossa patients; Table H. Baseline characteristics, by cohort and overall in supratentorial patients. (ZIP) [file pone.0178351.s004.zip › Table H.docx]

Table H: Baseline characteristics, by cohort and overall in supratentorial patients (n=148)†

| Characteristics | France  (n=17) | UK  (n=22) | Italy  (n=7) | GPOH HIT  (n=48) | Heidelberg  (n=54) | Total  (n=148) | p value* |
| --- | --- | --- | --- | --- | --- | --- | --- |
|  | N (%) | N (%) | N (%) | N (%) | N (%) | N (%) |  |
| Sex  Male  Female | 8 (47)  9 (53) | 9 (41)  13 (59) | 2 (29)  5 (71) | 30 (62)  18 (38) | 26 (48)  28 (52) | 75 (51)  73 (49) | 0.28 |
| Age at diagnosis (months)  median [Min - Max] | 83 [15-166] | 78 [4-182] | 47 [10-173] | 93 [3-223] | 72 [12-204] | 75 [3-223] | 0.83 |
| Age at diagnosis  <36months  ≥ 36 months | 6 (35)  11 (65) | 7 (32)  15 (68) | 3 (43)  4 (57) | 12 (25)  36 (75) | 10 (19)  44 (81) | 38 (26)  110 (74) | 0.43 |
| Grade  II  III  Missing | 3 (18)  14 (82) | 9 (41)  13 (59) | 2 (29)  5 (71) | 3 (6)  45 (94) | 7 (13)  47 (87) | 24 (16)  124 (84) | 0.006 |
| Extent of resection  Incomplete  Complete  Missing | 9 (53)  8 (47) | 10 (45)  12 (55) | 0 (0)  7 (100) | 16 (35)  30 (65)  2 | 25 (46)  29 (54) | 60 (41)  86 (59)  2 | 0.12 |
| Radiotherapy¥  No  Yes  Missing | 9 (53)  8 (47) | 7 (32)  15 (68) | 3 (43)  4 (57) | 2 (4)  45 (96)  1 | 18 (33)  36 (67) | 39 (27)  108 (73)  1 | 0.0004 |
|  | | | | | | |  |
| Tenascin-C  Negative  Positive | 13 (76)  4 (24) | 18 (82)  4 (18) | 4 (57)  3 (43) | 39 (81)  9 (19) | 30 (56)  24 (44) | 104 (70)  44 (30) | 0.03 |
| 1q25 gain  Negative  Positive | 13 (76)  4 (24) | 19 (86)  3 (14) | 7 (100)  0 (0) | 41 (85)  7 (15) | 48 (89)  6 (11) | 128 (86)  20 (14) | 0.64 |
| RELA  Negative  Positive  Missing | 6 (35)  11 (65) | 7 (70)  3 (30)  12 | 1 (20)  4 (80)  2 | 0 (0)  0 (0)  48 | 5 (13)  35 (88)  14 | 19 (26)  53 (74)  76 | <0.0001 |
|  | | | | | | |  |
| Median follow-up  [range] (years) | 6.0 [2.2;16.9] | 6.8  [1.6; 12.3] | 6.5  [3.3; 12.5] | 3.2  [0.0; 7.8] | 2.5  [0.3; 9.7] | 4.0  [0.0; 16.9] | <0.0001 |
| Number of death | 5 (29) | 4 (18) | 3 (43) | 5 (10) | 14 (26) | 31 (21) | 0.14 |
| Overall survival  median [95%CI] (years) | NA  [3.4 ; NA] | NA  [NA ; NA] | 12.5  [4.8 ; NA] | NA  [NA ; NA] | 6.3  [4.50 ; NA] | 12.5  [7.8 ; NA] | 0.22£ |
| Number of events | 11 (65) | 10 (45) | 4 (57) | 9 (19) | 24 (44) | 58 (39) | 0.005 |
| Event free survival  median [95%CI]  (years) | 1.8  [1.1 ; NA] | 9.5  [2.0 ; NA] | 2.5  [1.6 ; NA] | NA  [NA ; NA] | 3.1  [1.8 ; NA] | 6.8  [3.4 ; NA] | 0.05£ |

†: Patients with both TNC and 1q25 gain results; ¥: As no detailed information about treatment exist in Heidelberg cohort, we standardized treatment (None, chemotherapy alone, chemotherapy + radiotherapy and radiotherapy alone) in the other cohorts as radiotherapy (no, yes) in order to combine all cohorts. NA: Not assessable ; *: p-values were estimated using the Chi2, Kruskal-Wallis and logrank tests for binary, continuous and censored data, respectively; £ : indicates the p-value comparing the whole curve across countries
