# Supplementary material for: Integrating Tenascin-C protein expression and 1q25 copy number status in pediatric intracranial ependymoma prognostication: A new model for risk stratification
Source: PLoS One. 2017 Jun 15;12(6):e0178351. doi: 10.1371/journal.pone.0178351 (PMC5472261; doi:10.1371/journal.pone.0178351)
Supplement: S6 File — (PDF) [file pone.0178351.s006.pdf]

Reçu le 06.03.06

CNIL
Commission Nationale de l'Informatique et des Libertés

DUPLICATA du récépissé de déclaration du 12 septembre 2007

Numéro de déclaration
1176643

Madame Nicole BROUSSE
HOPITAL NECKER ENFANTS MALADES
119-149 RUE DE SEVRES
75743 PARIS CEDEX 15

Conformément à la loi du 6 janvier 1978 relative à l'informatique, aux fichiers et aux libertés, modifiée en août 2004,

HOPITAL NECKER ENFANTS MALADES
119-149 RUE DE SEVRES
75743 PARIS CEDEX 15

A déclaré à la Commission Nationale de l'Informatique et des Libertés un traitement automatisé d'informations nominatives dont la finalité principale est :

GESTION DE L'ENSEMBLE DES COLLECTIONS CRYOPRESERVEES ET DES DONNEES ASSOCIEES DE LA TUMOROTHEQUE DE L'HOPITAL

La délivrance du présent récépissé ne vaut pas constatation de la conformité du traitement à la loi et n'exonère le déclarant d'aucune de ses responsabilités.

Paris, le 03 septembre 2008
Par délégation de la commission

Alex Türk

Alex TÜRK
Président de la commission
